# New data on the aphid (Hemiptera, Aphididae) fauna of New Caledonia: some new biosecurity threats in a biodiversity hotspot 

**DOI:** 10.3897/zookeys.943.47785

**Published:** 2020-06-22

**Authors:** Christian Mille, Hervé Jourdan, Sylvie Cazères, Eric Maw, Robert Foottit

**Affiliations:** 1 IAC, Institut Agronomique néo-Calédonien, Équipe ARBOREAL AgricultuRe BiOdiveRsité Et vALorisation, Laboratoire d’Entomologie Appliquée, PO Box 32, 98880, La Foa, New Caledonia Institut Agronomique néo-Calédonien La Foa New Caledonia (Fr); 2 Institut Méditerranéen de Biodiversité et d’Écologie Marine et Continentale (IMBE), Aix-Marseille Université, UMR CNRS IRD Avignon Université, UMR 237 IRD, Centre IRD de Nouméa, PO Box A5, 98848, Nouméa Cedex, New Caledonia Aix-Marseille Université Nouméa Cedex New Caledonia (Fr); 3 Canadian National Collection of Insects, Agriculture and Agri-Food Canada/Agriculture et Agroalimentaire Canada Ottawa Research and Development Centre, K. W. Neatby Building 960 Carling Avenue, Ottawa, Ontario, K1a 0c6, Canada Agriculture and Agri-Food Canada/Agriculture et Agroalimentaire Canada Ottawa Research and Development Centre Ottawa Canada

**Keywords:** Aphids, biocontrol, biosecurity, invasive species, pests, Biosécurité, espèces envahissantes, lutte biologique, pucerons, ravageurs

## Abstract

Thirty-three species of aphids are now established in New Caledonia. All species appear to have been introduced accidentally by human activity in the last century. Here, 17 aphid species are recorded for the first time: *Aphiseugeniae*, *Aphisglycines*, *Aphisodinae*, *Aulacorthumsolani*, *Brachycaudushelichrysi*, *Cerataphisorchidearum*, *Greenideapsidii*, *Hyperomyzuscarduellinus*, *Hysteroneurasetariae*, *Lipaphispseudobrassicae*, *Micromyzuskatoi*, *Myzusornatus*, *Pentaloniacaladii*, *Rhopalosiphumnymphaeae*, *Rhopalosiphumrufiabdominale*, *Schizaphisrotundiventris*, and *Tetraneurafusiformis*. Thirteen more species are also more or less regularly intercepted at the borders through biosecurity surveys, without further establishment. This demonstrates that aphids represent a major biosecurity threat, including a threat as potential plant virus vectors. The reinforcement of biosecurity is a priority for such biodiversity hotspots, from the perspectives of both agriculture and the native environment. Prioritisation and promotion of local development of vegetable and fruit production, rather than their risky importation from abroad, is desirable. Such an approach also should be promoted and extended to other Pacific islands, which all share the lack of native aphid fauna and their associated plant disease vector risks.

## Abstract (fr)

Trente trois (33) espèces de pucerons sont aujourd’hui recensées de Nouvelle-Calédonie. Toutes ces espèces sont exotiques et ont été introduites accidentellement par les activités humaines. Dix-sept (17) espèces y sont ainsi recensées pour la première fois : *Aphiseugeniae*, *Aphisglycines*, *Aphisodinae*, *Aulacorthumsolani*, *Brachycaudushelichrysi*, *Cerataphisorchidearum*, *Greenideapsidii*, *Hyperomyzuscarduellinus*, *Hysteroneurasetariae*, *Lipaphispseudobrassicae*, *Micromyzuskatoi*, *Myzusornatus*, *Pentaloniacaladii*, *Rhopalosiphumnymphaeae*, *Rhopalosiphumrufiabdominale*, *Schizaphisrotundiventris* et *Tetraneurafusiformis*. Par ailleurs, au moins 13 autres espèces sont régulièrement interceptées par la biosécurité sans établissement actuel de populations. Les pucerons apparaissent donc comme une menace croissante pour la biosécurité de l’archipel. Aussi, le renforcement des mesures de biosécurité aux frontières apparaît prioritaire en association à une promotion du développement local de productions maraîchères et fruitières. Ainsi, la limitation de ces importations à risque, devrait contribuer à une meilleure protection des productions agricoles et de la biodiversité. Une telle approche devrait également être promue dans les pays insulaires du Pacifique, qui se caractérisent par la même disharmonie de peuplements, l’absence de communautés natives de pucerons et du risque associé de vectorisation de maladies phytopathogènes.

## Introduction

On a worldwide scale, aphids are currently represented by 5,558 valid species in 703 genera placed in 30 subfamilies (Favret 2018). Wegierek et al. (2017) state that aphids are known since the Permian, and appear more abundantly in the fossil records from the Early Cretaceous. But today, among this rich aphid fauna, only 250 species are considered as economically significant pests (Blackman and Eastop 2006; van Emden and Harrington 2007). Aphids are able to cause *direct damage* (through sap sucking and honeydew production) on all plant parts, and to cause *indirect damage* by transmission of plant viruses, which often has a greater impact on host plants. As they can be moved easily on commodities such as fresh fruits or ornamental plants, they are considered major quarantine insects on a world scale. Dissemination of exotic phytophagous insects among countries is an expected and significant side-effect of increased trade in fresh fruits, vegetables, and ornamental plants, and of tourist travel (Work et al. 2005; Hulme 2009). Establishment of exotic aphid species presents new threats to local agriculture (including the introduction of new plant viruses as aphids are well known as vectors). Adventive aphids also can result in significant restrictions in export trade (Batabyal and Beladi 2006; Dawson et al. 2017; Lohr et al. 2017; Turbelin et al. 2017). In this context, the continuation and expansion of international plant trade and human travel require sound and scientifically based phytosanitary protocols. Associated phytosanitary protocols rely on accurate and up-to-date pest species checklists, which are also essential for pest control research programs, especially for integrated pest management (IPM). Such lists also provide tools for biosecurity policies and managers (Charles and Henderson 2002; Beauvais et al. 2006). This is a particularly major issue for islands, where such introductions may have higher impacts and more serious ecological consequences, as they are depauperate of such pests and often have vacant ecological niches. The last list of aphids from New Caledonia was published in 1986 (Brun and Chazeau 1986) and an update was published by Jourdan and Mille (2006). The present checklist accounts for all encountered aphid species as well as known interceptions on fresh imported fruits and vegetables.

## Materials and methods

**Figure 1.**
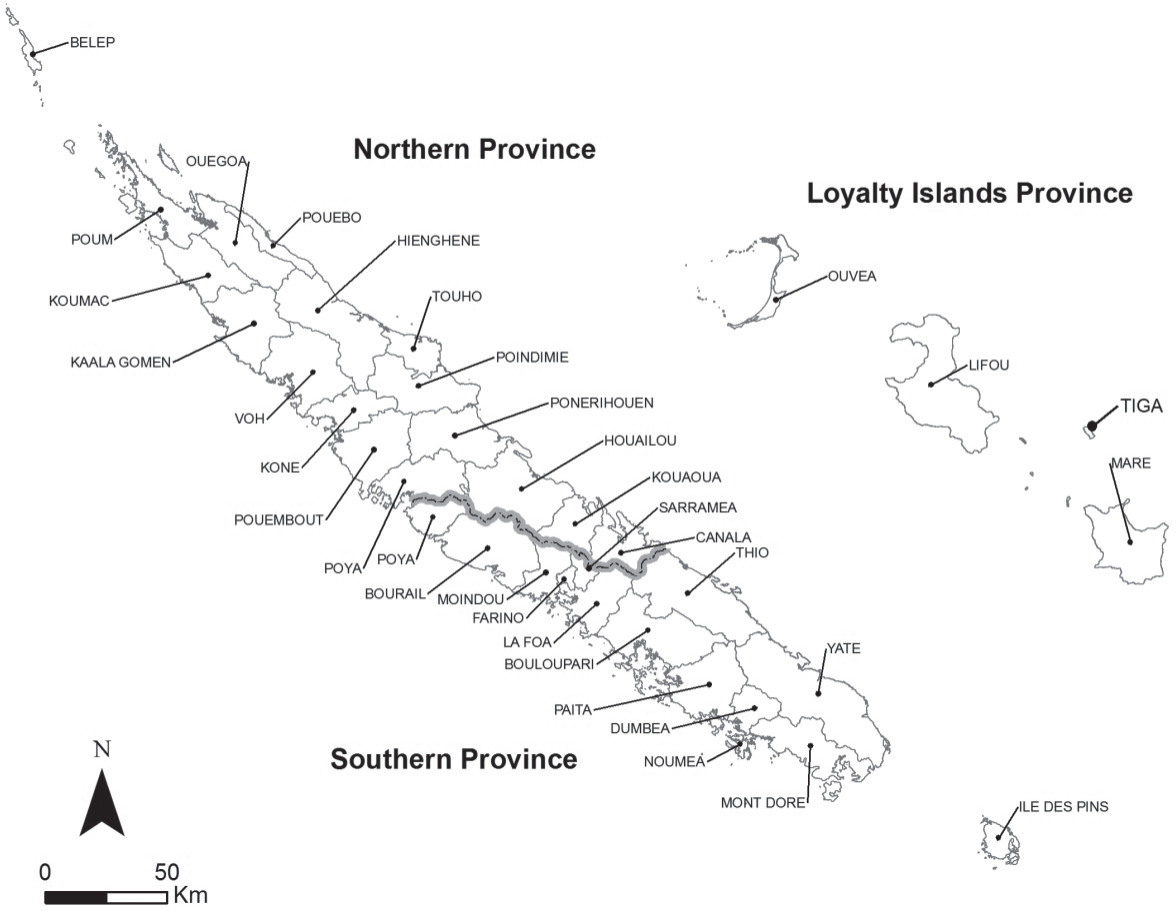
Map of New Caledonia showing administrative delimitations (provinces and counties).

The essential data of this work were compiled from scattered scientific literature and checklists, and from studies of curated specimens in the Collection de Référence des Invertébrés Terrestres de Nouvelle-Calédonie – Xavier Montrouzier, **CXMNC** (New Caledonia Terrestrial Invertebrate Reference Collection – Xavier Montrouzier), hosted at the Institut Agronomique néo-Calédonien (**IAC**, New Caledonian Agronomic Institute), and the identification of intercepted species to update the present list. Identifications were generally achieved by the late Mrs. Rosa C. Henderson (New Zealand Arthropod Collection, **NZAC**, Landcare Research, Auckland, New Zealand) and Mr. Eric Maw (Canadian National Collection of Insects, Arachnids & Nematodes, **CNC**, Canadian National Collection of Insects, Agriculture & Agri-Food Canada, Ottawa, Ontario, Canada). This updated and annotated checklist summarizes all recorded species from New Caledonia including the main island (Grande Terre) and adjacent inhabited islands; the Loyalty Islands (Lifu, Ouvéa, Maré, and Tiga), Belep Archipelago, and Isles of Pines (Figure 1). Currently valid species names are listed alphabetically, and subfamily and tribe are noted below each species. For each species, the original name of description, with author and year of description, are given. General geographic distributions are taken from the literature. Full synonymies are available on the Aphid Species File (Favret 2018). Literature records of the species in New Caledonia, host plants from literature records and local observations, biological control agents recorded in New Caledonia and observations on local distributions and economic importance are given.

**Abbreviations used**: coll. collector, det. determiner, dep. depositories.

## Results

Five tribes within four subfamilies are represented in New Caledonia: Aphidinae (Aphidini and Macrosiphini), Greenideinae (Greenideini), Hormaphidinae (Cerataphidini), and Eriosomatinae (Eriosomatini). Species names preceded by an asterisk are new records for New Caledonia.

### Current alphabetical aphid species list of New Caledonia

**Aphidinae**

**Aphidini**

**Aphis (Toxoptera) aurantii Boyer de Fonscolombe, 1841**

Black Citrus Aphid, Camelia Aphid, Puceron noir des Agrumes.

**Material examined.** On *Citrus* sp. (Rutaceae), in June 2000, R.C. Henderson det. (NZAC); La Foa County (IAC-SRFP), on leaves of *Citrus* sp. and same loc. on leaves of *Eugenia* sp. (Myrtaceae), 14.V.2003, S. Cazères coll., R.C. Henderson det. (NZAC); Sarraméa County (Réserve du Col d’Amieu) on unknown plant with orange and red young leaves, 18.X.2006, S. Cazères coll., R.C. Henderson det. (NZAC), dep. CXMNC; Tribe of Moméa, Moindou County, on unknown plant, 12.IV.2012, S. Cazères coll., E. Maw det. (CNC), dep. CXMNC; Yaté County (South of the Grande Terre) in Vale-Inco Plant Nursery, 21.IV.2016 on young plant of *Dodoneaviscosa* (L.) Jacq. (Sapindaceae), R.-M. M’Bouéri & C. Martin coll., E. Maw det. (CNC), dep. CXMNC.

**Remarks.** This species was first recorded by Cohic (1956) and Brun and Chazeau (1986) on *Citrus* spp.

It is distributed throughout the tropics and subtropics including Pacific islands, as well as in glasshouses in temperate climates (CABI 2019). This species is particularly important on citrus, cacao, coffee and tea, but also on sugar apple, fig, mango, ornamentals and some native plants (Blackman and Eastop 2000). Larval and adult ladybirds (Coccinellidae) such as *Coccinellatransversalis* (Fabricius, 1781) or *Menochilussexmaculatus* (Fabricius, 1781), both present in New Caledonia (Nattier et al. 2015), are known to feed on the species (Agarwala and Ghosh 1988; Roy and Rahman 2014). Aphidiinae wasps (Braconidae) like *Aphidiuscolemani* Vierek, 1912 present in New Caledonia can also parasitize this aphid (Kavallieratos et al. 2004).

This species is considered as a vector for the Citrustristeza (CTV)virus, but it is not a particularly efficient one. As regulatory measures already cover the protection of citrus, a strong surveillance is needed to prevent Aphis (Toxoptera) citricidus (Kirkaldy, 1907), the Tropical Citrus Aphid, from becoming established, as it is present in all Oceania around New Caledonia (CABI 2019). With *Aphisgossypii* (see below), *A.citricidus* is the most efficient vector of CTV (O’Connor 1969; Vogel 1978). The introduction of *A.citricidus* could compromise the ongoing eradication of CTV, which fortunately has not yet become pandemic in New Caledonia (Stéphane Lebegin, pers. comm. 13 January 2014). CTV was mostly spread by grafting of Washington Navel oranges (François Mademba-Sy, pers. comm. 7 March 2014), a cultivar imported with the pathogen from Australia during the late 1960’s. Most of the infected scions, with or without symptoms, which were distributed to the orchardists have now been destroyed, and CTV is considered as almost eradicated from New Caledonia. This statement is of course important for the New Caledonian citrus industry. But it is also significant for the conservation of biodiversity, as New Caledonia possess some early *Citrus* taxa (Bayer et al. 2009; Wu et al. 2018). The failure of the establishment of the CTV in New Caledonia could be explained by the presence of a “mild strain” of the virus, as suggested by some authors (e.g., Lee and Keremane 2013) and more likely to the absence of *A.citricidus*.

***Aphiscraccivora* Koch, 1854**

Groundnut Aphid, Puceron noir de la Luzerne.

**Material examined.** Tribe of Mucaweng, Lifu County (Loyalty Islands), 14.IV.2010 on an unknown leguminous plant in a forestry garden, H. Jourdan coll., dep. CXMNC; Pouembout County, 18.IV.2012 on *Solanumnigrum*, C. Mille coll., E. Maw det. (CNC), dep. CXMNC.

**Remarks.** This aphid was first recorded in New Caledonia as ‘*Aphisdolichis* Montrouzier, 1861’. Montrouzier (1861, p 74) found it in Lifu Island on a Fabaceae, a *Dolichos* which turned out to be *Vignaunguiculata* (L.) Walp., introduced from China. ‘*Aphisdolichis*’ was then synonymized with *A.craccivora* (Renaudière and Renaudière 1997). Bordat and Daly (1995) recorded this species from New Caledonia. An *Aphis* species was mentioned in Cohic (1956) and Brun and Chazeau’s (1986) catalogue which was probably in part *A.craccivora* based on the host plant species given: strawberry (*Fragariavesca* L.), tomatoes (*Solanumlycopersicum* L.), beans (*Phaseolus* spp.), garden peas (*Pisumsativum* L.), eggplants (*Solanummelongena* L.) and wheat (*Triticumaestivum* L.). *Aphiscraccivora* was also recently found on the European black nightshade, *Solanumnigrum* L. (Solanaceae).

Common in warm temperate and tropical regions, this highly polyphagous species can colonise young growths of numerous plants, mainly on Fabaceae, and including occasional records on Poaceae. It can be also found living on Araceae, Amaranthaceae, Asteraceae, Brassicaceae, Caryophyllaceae, Chenopodiaceae, Convolvulaceae, Cucurbitaceae, Cupressaceae, Ebenaceae, Euphorbiaceae, Lamiaceae, Lauraceae, Liliaceae, Malpighiaceae, Malvaceae, Moringaceae, Myrtaceae, Nyctaginaeae, Oleaceae, Orchidaceae, Pedaliaceae, Rubiaceae, Rutaceae, Sterculiaceae, and Zingiberaceae (CABI 2018).

Several natural enemies can control this species which is preyed by larvae and adults of various ladybirds (Coccinellidae) of which *Coccinellatransversalis*, *Harmoniaoctomaculata* (Fabricius, 1850) and *Menochilussexmaculatus* (Agarwala and Ghosh 1988; Sarma et al. 1996), present in New Caledonia (Nattier et al. 2015). This aphid is also known to be preyed by larvae of hoverflies (Syrphidae), especially *Ischiodonscutellaris* (Fabricius, 1805) (Sarma et al. 1996) and *Melanostomaunivittatum* (Wiedemann, 1824) both present in New Caledonia (Hull 1937). *Aphiscraccivora* is also known to be parasitized by aphidiine wasps (Braconidae) probably *Aphidiuscolemani*, which is the only species known to be present in New Caledonia, and is found on several aphid species (Starý 1975).

This species is a known vector of more than 30 plant viruses (Blackman and Eastop 1984) and must therefore be regarded as an important threat to New Caledonian crops.

*Aphiseugeniae van der Goot, 1917

**Material examined.** Mont-Dore County, Saint-Louis in IAC-SRMH, 23.II.2013, under the leaves of *Glochidionbillardieri* (Baill.) Müll. Arg. (Myrtaceae), G. Karnadi coll., E. Maw det. (CNC), dep. CXMNC.

**Remarks.** Originating from Southeast Asia, it is recorded eastward to Pakistan (Naumann-Etienne and Remaudière 1995), it is also known from Australia (Eastop 1966), Florida in 2011 (Skvarla et al. 2017) and Hawai’i (on Apocynaceae and Rosaceae; Foottit et al. 2012). It occurs most commonly on woody Euphorbiaceae, e.g., *Glochidion*, but has been recorded from plants in at least six other families (Blackman and Eastop 2006, 2020).

*Aphisglycines Matsumura, 1917

Soybean Aphid, Puceron du Soja.

**Material examined.** Boulouparis County (La Ouenghi) in an Adecal Technopole experimental plot, 23.II.2012, on *Glycinemax* (L.) Merr. (Soybean, Fabaceae), S. Cazères & C. Mille coll., E. Maw det. (CNC), dep. CXMNC.

**Remarks.** Originating from Asia, this almost cosmopolitan species is now present in the USA (Voegtlin et al. 2004), in Canada and in eastern Australia since 2000 (M. J. Fletcher, pers. comm. 2000). It is mainly on soybean and wild *Glycine* spp. and other Fabaceae.

Some ladybirds, of which *Harmoniaoctomaculata* and/or *Coccinellatransversalis*, appear to be very active against this aphid (personal observation in September 2012 by one of us, CM).

***Aphisgossypii* Glover, 1877**

Cotton Aphid, Melon Aphid, Puceron du Cotonnier.

**Material examined.** La Foa County (IAC-SRFP), on leaves of *Citrus* sp. 22.IV.2003, S. Cazères coll., R.C. Henderson det. (NZAC); same loc. on young leaves of *Psidiumguajava* L. (Myrtaceae), 1.IV.2004, S. Cazères coll., R.C. Henderson det. (NZAC); same loc. on *Cucurbitapepo* L. (Cucurbitaceae), 2.VIII.2006, J. Marin coll., R.C. Henderson det. (NZAC); same loc. on leaves of *Euphorbiahirta* L. (Euphorbiaceae), 29.XI.2008, C. Mille coll., R.C. Henderson det. (NZAC); Nouméa County, on unknown plant (round leaves), 21.I.2009, J. Marin coll., Rosa Henderson det. (NZAC); Nouméa County (Ouémo), on *Ocimumbasilicum* L. (Lamiaceae), 16.VI.12, M. Cazères coll., E. Maw det. (CNC), dep. CXMNC; Pouembout County, on Curcubitaceae, 11.VII.2013, C. Mille coll., E. Maw det. (CNC); Ouégoa County (North of the Grande Terre), on *Colocasiaesculenta* (L.) Schott leaves, 17.I.2014, E. Kastavi coll., E. Maw det. (CNC); La Foa County (Fonwhary), on *C.esculenta* leaves, 20.I.2014, L. Nemebreux coll., same loc. and same plant, 23.I.2013, S. Cazères & J. Brinon coll., E. Maw det. (CNC); Nouméa County (Ouémo), on *O.basilicum* leaves, 15.VI.2015, H. Jourdan coll., E. Maw det. (CNC); La Foa County (IAC-SRAP), on *O.basilicum* leaves, 3.VIII.2015, L. Marchal coll., E. Maw det. (CNC); Yaté County (South of the Grande Terre) in Vale-Inco Plant Nursery, on *Myodocarpusfraxinifolius* Brongn. & Gris (Myodocarpaceae), *Hibbertiapancheri* (Pancher & Sebert) Briquet (Dilleniaceae) and young plants of *Tarenna* sp. (Rubiaceae), 10.IX.2015, C. Mille coll., E. Maw det. (CNC), dep. CXMNC.

**Remarks.** Brun and Chazeau (1986) first recorded this species in New Caledonia. This highly polyphagous species is mainly found on Cucurbitaceae, Rutaceae, and Malvaceae. In addition to the plants given above, there are New Caledonian records from Apiaceae (*Apiumgraveolens* L., *Daucuscarota* L.), Apocynaceae (*Catharanthusroseus* (L.) G. Don), Araceae (*Alocasiamacrorrhizos* (L.) G. Don, *Caladiumbicolor* (Aiton) Vent., *Xanthosomasagittifolium* (L.) Schott), Asteraceae (*Dahlia* spp., *Leucanthemumvulgare* Lam.), Cucurbitaceae (*Citrulluslanatus* (Thunb.) Matsumura and Nakai, *Cucumis* spp., *Sechiumedule* (Jacq.) Swartz), Malvaceae (*Gossypium* spp., *Hibiscusrosa-sinensis* L.) and Rutaceae (*Citrus* spp.) (Brun and Chazeau 1986).

This cosmopolitan species is very common in the tropics and the Pacific region (Blackman and Eastop 2007). It is a major pest of cotton and cucurbits. *Aphisgossypii* transmits at least 76 plant viruses (Chan et al. 1991). Its natural enemies are larvae and adults of ladybirds (Coccinellidae) such as *Menochilussexmaculatus* and *Coccinellatransversalis* (Agarwala and Ghosh 1988). The ladybird *Diomusnotescens* (Blackburn, 1888) is known to prey on *Aphisgossypii* (Hopkinson et al. 2016) and also by *Micraspisfrenata* (Erichson, 1842) and *Coelophorainaequalis* (Fabricius, 1775), all three being present in New Caledonia (Nattier et al. 2015). It can be also controlled by hoverfly larvae (Syrphidae) and Aphidiinae wasps (Braconidae), especially *Aphidiuscolemani*.

This species regularly causes local heavy damage on various cultivated plants in New Caledonia and is therefore the most important pest aphid species in the country. This is also the main aphid species regularly surveyed for virus transmission in New Caledonia. *Aphisgossypii* also is considered as a good CTV vector (Cambra et al. 2000) although its efficiency is estimated between 6 and 25 times less effective than *Aphiscitricidus* (Halbert and Brown 1998). Thus, the occurrence of *Aphisgossypii* and *A.aurantii* in New Caledonia poses an important threat for New Caledonian citrus crops which represent 53% of all perennial fruit species grown in the country (Anonymous 2010), and to the ongoing CTV eradication program. However, preventing the establishment of *Aphiscitricidus* (see above, under *A.aurantii*) is the most important issue with regards to spread of CTV.

***Aphisnerii* Boyer de Fonscolombe, 1841**

Oleander Aphid, Puceron du Laurier rose.

**Material examined.** Farino County, 29.IV.2004 on *Asclepiasphysocarpus* Schlechter (Apocynaceae), S. Cazères coll., R.C. Henderson det. (NZAC).

**Remarks.** Cohic (1956) first recorded this species in New Caledonia, and Brun and Chazeau (1986) found it on the Tropical Milkweed, *Asclepiascurassavica* L. It was also collected on *Asclepiasphysocarpus* by one of us (SC).

Widely distributed through the tropical to warm temperate regions or subtropical areas including many Pacific islands. Its main hosts are Apocynaceae, especially *Neriumoleander* L., but it can also be found on Asteraceae, Convolvulaceae, Euphorbiaceae and Solanaceae. In Florida, it is occasionally observed on citrus (Rutaceae) without any damage (S. Halbert, pers. comm. 10 December 2019).

As for other aphids, predatory insects such as larvae and adults of the ladybirds (Coccinellidae) *Menochilussexmaculatus* (Agarwala and Ghosh 1988), the hoverfly larvae of *Ischiodonscutellaris* (Syrphidae) and lacewings of which two the two widespread species *Eumicromustasmaniae* (Walker, 1860) and *Malladabasalis* (Walker, 1853) (respectively Hemerobiidae and Chrysopidae) can control the populations in New Caledonia. *Aphidiuscolemani* wasps (Aphidiinae, Braconidae) are also known to parasitize the colonies of this aphid (Messing and Rabasse 1995).

The Oleander Aphid is able to transmit several viruses including SMV and PRSV which are respectively the Sugarcane mosaic potyvirus and the Papaya ringspot potyvirus (McAuslane 2017). However, the main concern with this species is its large and unsightly outbreaks on milkweeds. The damage caused by its colonies is mainly aesthetic due to the large amounts of sooty mould produced on plants.

*Aphisodinae (van der Goot, 1917)

Mango Aphid, Puceron du Manguier.

**Material examined.** On *Schinusterebinthifolius* Raddi (Anacardiaceae), 9.IX.2011, C. Mille coll., E. Maw det. (CNC), dep. CXMNC; Nouméa County 4.XI.2015, on *Mangiferaindica* L. (Anacardiaceae), F. Gimat coll., E. Maw det. (CNC).

**Remarks.** This species feeds on the undersides of leaves along main veins in dense colonies, attended by ants. It is commonly observed throughout the Old World tropics and subtropics on numerous plant species especially of the families Anacardiaceae, Araliaceae, Caprifoliaceae, Ericaceae, Rubiaceae, and Rutaceae. *Aphisodinae* is commonly grey-brown to rust-brown in colour, especially in Old World Tropics and in subtropics (Blackman et al. 2011) such as in New Caledonia. However, some much darker forms occur in Asia and a dark green form is found in Japan (Blackman et al. 2011). It has not yet been implicated in the transmission of any plant virus (Blackman and Eastop 1984).

***Aphisspiraecola* Patch, 1914**

Spirea Aphid, Green Citrus Aphid, Puceron des Spirées.

**Material examined.** Mont-Dore County (Saint-Louis) in IAC-SRA, 3.VIII.2004 on *Pittosporumcoccineum* (Montrouz.) Beauvis. (Pittosporaceae), G. Gâteblé coll., R.C. Henderson det. (NZAC), dep. CXMNC; same loc. 31.I.2006 on *Artiabalansae* (Baill.) Pichon (Apocynaceae), G. Gâteblé coll., R.C. Henderson det. (NZAC); same loc. 7.VIII.2013 on *Ixoracauliflora* Montrouz. (Rubiaceae), E. Maw det. (CNC).

**Remarks.** This polyphagous species was first recorded in New Caledonia on *Citrus* spp. by Jourdan and Mille (2006). It is recorded from Araceae, Araliaceae, Convolvulaceae, Fabaceae, Lythraceae, Magnoliaceae, Nyctaginaeceae, Rutaceae, Solanaceae and Verbanaceae, but occurs especially on Asteraceae, Caprifoliaceae, and Rosaceae. It has an almost cosmopolitan distribution.

Most predatory insects of this aphid are the adults and larvae of the ladybird *Harmoniaoctomaculata* (Coccinellidae) and the larvae of the hoverfly *Ischiodonscutellaris* (Syrphidae). Also, *Aphidiuscolemani* (Braconidae, Aphidiinae) parasitizes this aphid (Tomanović et al. 2009).

In many countries, the Green Citrus Aphid is the most damaging species to the citrus fruit industry. In addition to direct damage and the production of honeydew, which favors the development of sooty moulds, this pest constitutes also a potential vector of the CTV (Kalaitzaki et al. 2019).

**Hormaphidinae**

**Cerataphidini**

***Astegopteryxbambusae* (Buckton, 1893)**

Bamboo leaf Aphid, Puceron des feuilles du Bambou.

**Remarks.** This species was first recorded in New Caledonia by Dr Paul Cochereau in the 60’s on *Bambusa* spp. (Brun and Chazeau 1986). *Astegopteryxbambusae* occurs throughout East and South-East Asia, generally colonising the undersides of the leaves of bamboos.

Known natural enemies are the coccinellids *Anisolemniadilatata* (Fabricius) and *Synonychagrandis* (Thunberg), both absent from New Caledonia (Nattier et al. 2015).

**Aphidinae**

**Macrosiphini**

*Aulacorthumsolani (Kaltenbach, 1843)

Foxglove Aphid, Puceron à taches vertes de la Pomme de terre.

**Material examined.** La Foa County (IAC-SRFP), 11.V.2007 on leaves and fruits of *Capsicumannuum* L. (Solanaceae), P. Caplong coll.; Poindimié County (Wagap), 17.VII.2007 on *Ipomoeabatatas* (L.) Lam. (Convolvulaceae), D. Varin coll., both identified by R.C. Henderson det. (NZAC), dep. CXMNC.

**Remarks.** This cosmopolitan and very polyphagous aphid is present on many different families of plants and it is a common pest in glasshouses. *Aulacorthumsolani* transmits at least 45 plant viruses (Chan et al. 1991). Further investigations of this species in New Caledonia are needed with respect to plant virus transmission.

*Brachycaudushelichrysi (Kaltenbach, 1843)

Leaf-curling Plum Aphid, Puceron vert du Prunier.

**Material examined.** La Foa County (IAC-SRFP), 16.VI.2015 on leaves of *Ageratumconyzoides* L. (Asteraceae), S. Cazères coll., E. Maw det. (CNC), dep. CXMNC.

**Remarks.** Today, this species is globally distributed. Its primary hosts are *Prunus* spp. (Rosaceae), and its secondary hosts are numerous species of Asteraceae, Boraginaceae and sometimes Fabaceae, as well as many ornamental plants.

The only ladybird cited to feed on this aphid and present in New Caledonia is *Menochilussexmaculatus* (Agarwala and Ghosh 1988). Regarding syrphid flies, *Melanostomaunivittatum*, present in New Caledonia (Hull 1937), is known to prey on this aphid. This aphid is also parasitized by *Aphidiuscolemani*, also present in New Caledonia (Starý 1975).

It is involved in the transmission of several plant viruses, including the Cucumber mosaic virus (Blackman and Eastop 1984).

***Brevicorynebrassicae* (Linnaeus, 1758)**

Cabbage Aphid, Puceron cendré du Chou.

**Remarks.** Restricted to the members of the Brassicaceae, Cohic (1956) and Brun and Chazeau (1986) recorded this species in New Caledonia on cabbage (*Brassica* spp.) and on radish (*Raphanussativus* L.). This species is distributed in all temperate and warm parts of the world.

As predators of this aphid species, Agarwala and Ghosh (1988) cite several ladybird species but the only one present in New Caledonia is *Coccinellatransversalis* (Coccinellidae). Joshi and Ballal (2013) indicate that *Ischiodonscutellaris* is a good predator of this aphid. *Diaeretiellarapae* (M’Intosh, 1855) (Braconidae, Aphidiinae) is known to parasitize *B.brassicae* but is absent from New Caledonia (Lopez et al. 2016); this beneficial species could be a good candidate to enhance the biological control of this aphid.

It is an important pest on Brassicaceae and has been involved in the transmission of at least 20 plant viruses (Blackman and Eastop 1984).

***Capitophoruselaeagni* (del Guercio, 1894)**

Artichoke Aphid, Puceron vert de l’Artichaut.

**Remarks.** Brun and Chazeau (1986) first recorded this species in New Caledonia on the Artichoke thistle (*Cynaracardunculus* L.) and on the Barberton daisy (*Gerbera* spp.), both Asteraceae. Widely distributed through the temperate and warm temperate regions of the world (Blackman and Eastop 2000). The populations are mainly on undersides of leaves, but some are also observed on upper sides of young leaves. It is also reported on Polygonaceae.

Some entomopathogenic fungi can limit the importance of the colonies (Jouda et al. 2010).

**Hormaphidinae**

**Cerataphidini**

***Cerataphislataniae* (Boisduval, 1867)**

Latania Aphid, Puceron du Latanier.

**Remarks.** Brun and Chazeau (1986) first recorded this species in New Caledonia only on palms of *Cocosnucifera* (Arecaceae). Outside of tropical regions, *Cerataphislataniae* is known in most of Europe (*Areca* and *Musa* spp., respectively Arecaceae and Musaceae), in Asia region, in North, Central and South America and Oceania. It appears to be widespread on palms, especially *Latania* spp. and other fan-palms, *Raphia* spp. and the coconut tree, through the tropics and in glasshouses. Pérez Hidalgo et al. (2000) signal this species as introduced in the Canary Isles, where it also colonizes *Strelitziaalba* (L.f.) Skeels (Strelitziaceae).

This species is not a major phytosanitary problem, but it has spread globally.

However, there is much confusion in the literature between this species and its close relative *Cerataphisbrasilensis* (Hempel, 1901), which is also widely distributed and colonises various palms including coconuts, so the identity of the species in New Caledonia needs further verification.

*Cerataphisorchidearum (Westwood, 1879)

Orchid Aphid, Puceron des Orchidées.

**Material examined.** Lifu Island County (Loyalty Islands), 23.III.2012 on *Vanilla* sp. (Orchidaceae), J-P. Lolo coll., E. Maw det. (CNC), dep. CXMNC; Maré Island County (Loyalty Islands), 6.VIII.2012 on *Vanilla* sp., C. Mille coll., det. S. Cazères, dep. CXMNC; Maré Island County (Loyalty Islands), 3.IV.2013 on *Vanilla* sp., J. Drouin coll., det. S. Cazères, dep. CXMNC.

**Remarks.** This pantropical species is found on various Orchidaceae in the tropics, and in European and North American glasshouses.

**Greenideinae**

**Greenideini**

*Greenideapsidii van der Goot, 1917

Asian Guava Aphid, Puceron asiatique du Goyavier.

**Material examined.** Tribe of Moméa, Moindou County, 12.IV.2012 by beating an unknown myrtle plant (Myrtaceae), S. Cazères coll., E. Maw det. (CNC), dep. CXMNC.

**Remarks.** This aphid is an invasive pest that feeds on young shoots and undersides of young leaves of ecologically and economically important plants of the family Myrtaceae: *Psidiumguajava* L., *Rhodomyrtus* spp., *Eugenia* spp., *Melaleuca* spp., *Plinia* spp. Originating from the Indo-Asian region, this species is now widely distributed in temperate and tropical regions, including Australia in the vicinity of New Caledonia (Blackman and Eastop 1994). It is also reported in Hawai’i (Beardsley 1993).

Potential natural enemies of this invasive aphid include Chrysopidae, Coccinellidae and Braconidae (Culik et al. 2016).

**Aphidinae**

**Macrosiphini**

*Hyperomyzuscarduellinus (Theobald, 1915)

Asian Sowthistle Aphid.

**Material examined.** Tribe of Hnae, Tiga (Loyalty Islands), 11.IV.2017, collected on *Sonchusoleraceus* L. (Asteraceae) in a garden, R.-M. M’Bouéri coll., E. Maw det. (CNC).

**Remarks.** This species is widely distributed in warm temperate and subtropical parts of the world including Australia, Fiji Islands and Hawai’i, and colonises many genera of Asteraceae.

The fungus *Pandoraneoaphidis* is known to infect up to 70% of *Hyperomyzuscarduellinus* populations in Argentina (Manfrino et al. 2013) but is absent from New Caledonia.

***Hyperomyzuslactucae* (Linnaeus, 1758)**

Currant-sowthistle Aphid, Puceron des feuilles du Groseillier et de la Laitue.

**Remarks.** Brun and Chazeau (1986) first recorded this species in New Caledonia on *Sonchus* spp., (Asteraceae). It is now distributed all over the world except Southern Africa, feeding on new shoots and undersides of young leaves of *Sonchus* spp., which curl slightly and show yellow spots. It is occasionally found on other Asteraceae.

The ladybird *Coelophoramulsanti* is known to prey on this aphid in New Caledonia (Sallée and Chazeau 1985). There are no New Caledonian records of hoverflies (Diptera, Syrphidae) or hymenopterous parasitoids attacking this aphid.

It is the vector of approximately 12 non-persistent viruses (Boakye and Randles 1974).

**Aphidinae**

**Aphidini**

*Hysteroneurasetariae (Thomas, 1878)

Rusty Plum Aphid, Puceron brun du Prunier.

**Material examined.** Nouméa County in PANC (Port Autonome de Nouvelle-Calédonie), 19.V.2015, on *Paspalumdigitatum* (Sw.) Kunth (Poaceae), F. Gimat coll., E. Maw det. (CNC), dep. CXMNC; same loc. on an unknown plant, 7.III.2016, L. Sariman coll., E. Maw det. (CNC), dep. CXMNC.

**Remarks.** Detected on *Paspalumdigitatum* in large numbers in New Caledonia, elsewhere it is also known on many other Poaceae species such as rice (*Oryzasativa* L.), sugarcane (*Saccharumofficinarum* L.), *Sorghum* spp. and some species of Cyperaceae. It is native of North America but is now distributed in many countries and regions of the world after a rapid spread as in Europe (Coeur d’acier et al. 2010). It is also present in regions near New Caledonia, such as Australia, Fiji Islands, Papua New Guinea, Solomon Islands, and Indonesia (Nasruddin 2013).

If this newly arrived species becomes a pest, larvae and adults of ladybirds (Coccinellidae) present in New Caledonia such as *Coccinellatransversalis* or *Menochilussexmaculatus* (Nattier et al. 2015) are known to feed on this species and are used in banker plant systems to maintain some predator populations near to crops for protection (Rattanapun 2017).

**Aphidinae**

**Macrosiphini**

*Lipaphispseudobrassicae (Davis, 1914)

Turnip Aphid, Puceron de la Moutarde.

**Material examined.** La Foa County (Nili), 16.VIII.2011 on “rocket” (*Erucasativa* Mill., Brassicaceae) on a hydroponic kit, C. Mille coll., E. Maw det. (CNC), dep. CXMNC.

**Remarks.** The Turnip Aphid is also recorded on many genera and species of Brassicaceae and is widespread in the world (Blackman and Eastop 2000).

***Macrosiphumeuphorbiae* (Thomas, 1878)**

Potato Aphid, Puceron vert et rose de la Pomme de terre.

**Remarks.** Cohic (1958a) first recorded this species in New Caledonia on tomato (*Solanumlycopersicum* L., Solanaceae). This cosmopolitan and polyphagous species feeds on over 200 plant species and can transmit at least 67 plant viruses (Chan et al. 1991). It should be monitored closely in New Caledonia because of its potential to become a serious pest.

The known beneficial agents against this aphid are not recorded from New Caledonia, but some of the present ones probably play an important role in its control. Some entomopathogenic fungi have also shown some promising clues for the biological control in greenhouses (Fournier and Brodeur 1999).

Cohic (1958a) rated this species as a very important pest on tomato during dry seasons, but there is no current information. The most dangerous activity of this aphid is the transmission of phytopathogenic viruses, especially the Potato Y virus (PYV) and the Beet yellow virus (BYV). Fortunately, the Potato Aphid does not transmit the Tomato yellow leafcurl virus (TYLCV) recently detected in New Caledonia (Péréfarres et al. 2012).

***Macrosiphumrosae* (Linnaeus, 1758)**

Rose Aphid, Puceron vert du Rosier.

**Material examined.** Bourail County (Gouaro), 19.X.2006 on young leaves of *Rosa* spp. S. Cazères coll., E. Maw det. (CNC), dep. CXMNC.

**Remarks.** Cohic (1956) and Brun and Chazeau (1986) first recorded this species in New Caledonia on *Rosa* spp. (Rosaceae). This species is widespread in most of the world on cultivated roses, except Japan and southeast Asia. Secondary hosts are Dipsacaceae and Valerianaceae. Blackman and Eastop (2000) also recorded it on other Rosaceae (*Fragaria* spp., *Geum* spp., *Pyrus* spp., *Malus* spp., *Rubus* spp.) and on Onagraceae (*Chamaenerion* spp., *Epilobium* spp.).

In New Caledonia, the known present beneficial agents are *Coccinellatransversalis* (Coccinellidae), *Eumicromustasmaniae* (Neuroptera, Hemerobiidae) and the entomopathogenic fungus *Lecanicilliumlecanii*.

This species is able to transmit at least 12 plant viruses including the persistent Strawberry mild yellow edge virus and should therefore be regularly checked in the New Caledonian context, but it is not a vector of the Rose mosaic virus (Blackman and Eastop 1984).

*Micromyzuskatoi (Takahashi, 1925)

**Material examined.** Lifu Island County (Loyalty Islands), 14.IV.2010, on ferns (Polypodiaceae) in an agro-forestry garden, H. Jourdan coll., E. Maw det. (CNC), dep. CXMNC.

**Remarks.** This species is recorded from Australia, Hawai’i, Indonesia, and Taiwan. It is observed on undersides of fronds of several genera of Polypodiaceae ferns of the genera *Microsorum*, *Platycerium*, and *Polypodium*.

*Myzusornatus Laing, 1932

Ornate Aphid, Puceron orné.

**Material examined.** La Foa County (Nili), 1.VII.2015 on the Liliaceae*Lilium* sp. (orange flower), Z. Lemerre Desprez coll., E. Maw det. (CNC), dep. CXMNC.

**Remarks.***Myzusornatus* is widespread throughout the world, probably because of commercial trade in ornamental plants. It is a very polyphagous species. Besides the Liliaceae, it infests plant species in Apiaceae, Asteraceae, Bignoniaceae, Brassicaceae, Caryophyllaceae, Lamiaceae, Polygonaceae, Primulaceae, Rosaceae, Solanaceae and Violaceae.

It is regarded as a pest of various plants because it transmits at least 20 plant viruses, including Potato leaf roll virus.

***Myzuspersicae* (Sulzer, 1776)**

Green Peach Aphid, Puceron vert du Pêcher.

**Material examined.** La Foa County (Pocquereux), 31.VIII.2015 on *Solanumtuberosum* L. (Red Pascal cv, Solanaceae), N. Hugot coll., E. Maw det. (CNC), dep. CXMNC; La Foa County, 15.IX.2015 on *Solanumtuberosum*, N. Hugot coll., E. Maw det. (CNC); Dumbéa County at Nondoué Farm, 12.IX.2016 on *Brassicaoleracea* L. (Brassicaceae), C. Mille coll., E. Maw det. (CNC).

**Remarks.** Cohic (1956) and Brun and Chazeau (1986) first recorded this species in New Caledonia on *Brassica* spp., *Citrus* spp., *Prunuspersica*, and *Solanummelongena* (respectively Brassicaceae, Rutaceae, Rosaceae, and Solanaceae). This almost cosmopolitan species is highly polyphagous, recorded from more than 40 plant families.

Sharanabasappa et al. (2007) have shown the potential of predation of this aphid by the hoverfly *Ischiodonscutellaris*. The two species of ladybirds cited as predators of *M.persicae* by Agarwala and Ghosh (1988) and present in New Caledonia are *Harmoniaoctomaculata* and *Menochilussexmaculatus* (Nattier et al. 2015). *Aphidiuscolemani* (Braconidae, Aphidiinae) is known to be a very effective parasitoid against *M.persicae* (Messing and Rabasse 1995). Some predatory midges (Cecidomyiidae) have been collected in 2000 but without a formal identification to date. In greenhouse-grown vegetables in Europe, there has been considerable success using the entomopathogenic fungus *Verticillium* sp. (Mackauer 1968).

The Green Peach Aphid is the most important virus vector as it is able to transmit at least 182–200 plant viruses (Kennedy et al. 1962; Chan et al. 1991). In 2017, this species was the most intercepted aphid species, with 89 specimens among 192 intercepted aphids during 20 events of interceptions on fresh fruits and vegetables imported into New Caledonia (Cazères and Mille 2018).

*Pentaloniacaladii van der Goot, 1917

Cardamom Aphid, Puceron de la Cardamome.

**Material examined.** Dumbéa County at Nondoué Farm, 12.IX.2016 on *Colocasiaesculenta* (L.) Schott (Araceae), C. Mille coll., E. Maw det. (CNC).

**Remarks.***Pentaloniacaladii* was discovered in New Caledonia following the advice of Ross Miller of the University of Guam (R. Miller, pers. comm. 10 December 2010). This species is found on plants in the families of Zingiberaceae and Araceae, or occasionally on plants in other families (Heliconiaceae, Musaceae). It is widely distributed throughout the South Pacific, and is also known in China (Hong Kong), Australia and in glasshouses of the Northern Hemisphere.

Its known natural enemies are ladybird larvae and adults (Coccinellidae) and hoverfly larvae (Syrphidae).

It is known to be a Banana bunchy top virus (BBTV) vector (Watanabe et al. 2013). These authors add that the role played by *P.caladii* in the BBTV transmission would need some further studies. This species has been long regarded as a synonym of *P.nigronervosa*, or as a “form” of that species, but molecular and multivariate studies by Foottit et al. (2010) have shown that it should be treated as a distinct species.

***Pentalonianigronervosa* Coquerel, 1859**

Banana Aphid, Puceron noir du Bananier.

**Material examined.** Poindimié County (North East of Grande Terre), 28.XI.2011 on *Musa* sp. (Musaceae), I. Murcia coll., R.C. Henderson det. (NZAC), dep. CXMNC; Dumbéa County at Nondoué Farm, 12.IX.2016 on *Musa* sp., C. Mille coll., E. Maw det. (CNC).

**Remarks.** It was recorded officially by Jourdan and Mille (2006) but was first identified in 1991 by François Leclant from specimens collected in La Foa County (Pocquereux Valley). This pantropical species is widespread through all tropical and subtropical parts of the world, and is introduced into glasshouses in Europe and North America. It occurs on other members of the families Musaceae (*Musa* spp.) and possibly Heliconiaceae (*Heliconia* spp.), although some records may be due to past confusion with *P.caladii*.

This relatively recent arrival is unexpected, as banana trees have been moved around in the Pacific Region for the last 3,000 years. It only took ten years after the discovery of the aphid for the outbreak of the BBTV that was not detected in the original population of banana aphids. This sad example illustrates the high importance of sound quarantine policy regarding allowance of plants that might arbor viruses that are absent, when a potential vector already is present. The importance of this species in New Caledonia dramatically increased in 2001 with the discovery of BBTV (Kagy et al. 2001). It also is able to transmit Banana mosaic, Abaca bunchy top, and Cardamom mosaic viruses. Importantly, it is the sole vector of BBTV in Australia, Africa and Asia. The aphids can be found living under the old leaf bases, sometimes ant-attended (Blackman and Eastop 2000). *Pentalonianigronervosa* was not recorded in Brun and Chazeau’s catalogue (1986). It was identified formally in 1991 in the Pocquereux Fruit Research Station (Mille 2000). Prior to 1999, the date of the discovery of the BBTV in New Caledonia (Kagy et al. 2001), *P.nigronervosa* was not significant to banana crops, but then it became an important pest, as it was partly responsible of the spread of the BBTV throughout the country.

**Aphidinae**

**Aphidini**

***Rhopalosiphummaidis* (Fitch, 1856)**

Maize Aphid, Corn Leaf Aphid, Puceron vert du Maïs.

**Material examined.** Boulouparis County (La Ouenghi) in an Adecal Technopole experimental plot, 23.II.2012, on maize (*Zeamays* L., Poaceae), S. Cazères & C. Mille coll., E. Maw det. (CNC), dep. CXMNC.

**Remarks.** Brun and Chazeau (1986) first recorded this species in New Caledonia on the Poaceae*Sorghumbicolor* (L.) Moench and maize (*Zeamays* L.). This cosmopolitan species is found on young leaves of grasses including the genera *Avena*, *Hordeum*, *Oryza*, *Saccharum*, *Secale*, *Sorghum*, *Triticum*, *Zea*, and occasionally Cyperaceae and Typhaceae (Blackman and Eastop 2000).

This aphid is preyed on by larval and adult ladybird species *Coccinellatransversalis* and *Menochilussexmaculatus* (Coccinellidae) (Agarwala and Ghosh 1988), and by the hoverfly larvae *Ischiodonscutellaris* (Syrphidae) (Ghorpadé 1981). It is also parasitized by the wasp *Aphidiuscolemani* (Braconidae, Aphidiinae).

This is probably the most important pest of cereals in tropical and warm climates because it can transmit the pathogens in the yellow dwarf virus complex and at least five other viruses (Blackman and Eastop 2000). It causes a longitudinally rolling of the last leaf during growth and secretes abundant honeydew on which sooty mould develops.

*Rhopalosiphumnymphaeae (Linnaeus, 1761)

Water Lily Aphid, Puceron noir du Nénuphar.

**Material examined.** Mont-Dore County (Saint-Louis) in IAC-SRMH/Biofabrique D.D.R. (Beneficials rearing factory, Direction du Développement Rural, Southern Province), 12.XI.2013 and 5.XII.2013 on the invasive waterfern *Salviniamolesta* D. S. Mitch. (Salviniaceae), B. Gatimel coll., E. Maw det. (CNC); Bourail County (Gouaro), 28.III.2016 on *Nymphaea* sp. (purple flower, Nymphaeaceae), S. Cazères coll., E. Maw det. (CNC).

**Remarks.** This almost cosmopolitan species forms colonies which occur on a large variety of water plant genera as *Alisma*, *Butomus*, *Callitriche*, *Echinodorus*, *Juncus*, *Nelumbo*, *Nuphar*, *Nymphaea*, *Potamogeton*, *Sagittaria*, *Sparganium*, *Triglochin*, *Typha*, etc. Its primary hosts are *Prunus* spp. (Rosaceae), but in the tropics it is probably entirely anholocyclic.

The two genera *Coccinella* and *Harmonia* represented in New Caledonia (Nattier et al. 2015) are known to prey on this aphid species (Agarwala and Ghosh 1988), which is also parasitised by *Aphidiuscolemani* (Braconidae, Aphidiinae) (Tomanović et al. 2012).

*Rhopalosiphumnymphaeae* has been used for biological control of water weeds in rice plots (Oraze and Grigarick 1992).

***Rhopalosiphumpadi* (Linnaeus, 1758)**

Bird Cherry-Oat Aphid, Puceron du Merisier à grappes.

**Remarks.** Brun and Chazeau (1986) first recorded this species in New Caledonia living on the two Poaceae species *Sorghumbicolor* (L.) Moench (“*Sorghumvulgare*”) and maize (*Zeamays* L.). This species is now virtually cosmopolitan. Primary hosts are *Prunus* spp. and secondary hosts are numerous species of Poaceae, including all the major cereals and pasture grasses.

Sallée and Chazeau (1985) studied the New Caledonian endemic ladybird *Coelophoramulsanti* (Montrouzier, 1861) (Coleoptera, Coccinellidae) as a predator of *Rhopalosiphumpadi*. *Aphidiuscolemani* is also known to control this aphid species (Elliott et al. 1994; Hullé et al. 2006).

This aphid is known to transmit pathogens in the yellow dwarf virus complex (D’Arcy et al. 1981), which is absent from New Caledonia, but present in Australia and New Zealand (Smith 1964).

*Rhopalosiphumrufiabdominale (Sasaki, 1899)

Rice Root Aphid, Red Rice Root Aphid.

**Material examined.** Tribe of Mou, Lifu Island (Loyalty Islands), 03.XI.2017, on roots of some hydroponic lettuce (*Lactucasativa* L., Asteraceae) D. Pastou coll., E. Maw det. (CNC).

**Remarks.** This species appears to be Oriental and was first described from Japan, but it is now almost cosmopolitan. Remaudière and Etienne (1988) documented its presence on Réunion Island, which could explain a potential pathway of the species for its arrival in New Caledonia. This pathway is already observed for some scale insects (Mille et al. 2016a). It is known to be on underground parts of numerous species of Poaceae (sugarcane, oats, barley, millet, and wheat), Cyperaceae and some dicotyledons, particularly Solanaceae (eggplant, potato, tomato, tobacco and capsicum), also marrow and cotton.

The entomopathogenic fungus *Verticilliumlecanii*, known to be present in New Caledonia (Mille 2011; Mille et al. 2016a), has been recorded on this aphid (Etzel and Petitt 1992). Some predators and parasitoids are cited (Yano et al. 1983) but do not seem to be efficient against this species.

The Rice Root Aphid has a very broad host plant range, having been recorded from 22 plant families. Like the previous aphid species, this one uses *Prunus* spp. (Rosaceae) as primary hosts in east Asia (Blackman and Eastop 2000) and in Southern Europe (Rakauskas et al. 2015). It is known to be a vector of Barley yellow dwarf virus (Paliwal 1980), Cereal yellow dwarf virus (Hadi et al. 2011), Maize mosaic virus in India (Singh 1977), and Sugarcane mosaic virus also in India (Shukla and Sinha 2009). It is thought to be a non-persistent vector of the Cucumber mosaic virus, causing serious damages on tobacco in Taiwan (Chen and Weng 1969). The present development of grain crops, especially rice and wheat, in New Caledonia, in order to minimize imports, could be threatened by these viruses. Strong phytosanitary regulations are needed to avoid their introduction.

*Schizaphisrotundiventris (Signoret, 1860)

Oil Palm Aphid.

**Material examined.** Tribe of Hwadrilla, Ouvéa Island (Loyalty Islands), 13.III.017, from Winkler traps in a garden, E. Bourguet coll., E. Maw det. (CNC).

**Remarks.** This species is considered nearly cosmopolitan (Skvarla et al. 2018). Its origin is uncertain, but sexual forms occur on pear trees (*Pyruscommunis*) on the southern flanks of the Himalayan Mountains (Naumann-Etienne and Remaudière 1995). In other parts of the world *Schizaphisrotundiventris* lives all year around on secondary hosts, mainly on Cyperaceae but sometimes on other monocotyledons (Remaudière and Etienne 1988).

*Toxaresmacrosiphophagum* Shuja-Uddin, 1974 (Hymenoptera, Braconidae, Aphidiinae) and an unknown species of *Aphidius* are known from India on this aphid (Starý and Ghosh 1983). These two species could be candidates for the development of a biological control measure if *S.rotundiventris* becomes a pest in New Caledonia.

As this species was caught in Winklers traps during an invasive insect survey in the Loyalty Islands, its presence on the Grande Terre should be investigated.

**Eriosomatinae**

**Eriosomatini**

*Tetraneurafusiformis Matsumura, 1917

Root Aphid, Puceron des racines.

**Material examined.** Mont-Dore County, Rivière des Pirogues (South of the Grande Terre) at the Champalou Farm, 8.V.2007 on the roots of *Paspalumpaniculatum* L. (Poaceae), P. Caplong coll., R.C. Henderson det. (NZAC), dep. CXMNC.

**Remarks.** It is also recorded in Africa, Central and South America, Australia, Fiji Islands, South and East Asia and Tonga (Foottit et al. 2012). *Tetraneurafusiformis* is known in colonies on roots of many genera and species of Poaceae (*Agropyron*, *Axonopus*, *Cenchrus*, *Chloris*, *Cynodon*, *Dactyloctenium*, *Echinochloa*, *Eleusine*, *Eragrostis*, *Oryza*, *Panicum*, *Paspalum*, *Pennisetum*, *Saccharum*, *Setaria*, *Sorghum*). Its presence is often indicated by a reddish-purple discoloration of the leaves.

We have recorded 33 species from New Caledonia. To date, all these species appear exotic to the archipelago. Among them, 17 are formally recorded for the first time in New Caledonia.

An overview of all species is compilated in Table 1. The aphid species recorded from endemic New Caledonian plants are listed in Table 2, and Table 3 summarizes information about the beneficial species mentioned in the text.

**Table 1.** List of the 33 aphid species in New Caledonia. First records are in bold.

Subfamilies	Tribes	Species	Biogeographic region of origin and record
Aphidinae	Aphidini	*Aphisaurantii* (Boyer de Fonscolombe, 1841)	Probably Oriental, Brun and Chazeau (1986)
		*Aphiscraccivora* Koch, 1854	Palaearctic, Bordat and Daly (1995), Jourdan and Mille (2006)
		***Aphiseugeniae* van der Goot, 1917**	**Oriental**
		***Aphisglycines* Matsumura, 1917**	**Oriental**
		*Aphisgossypii* Glover, 1877	Oriental, Brun and Chazeau (1986)
		*Aphisnerii* Boyer de Fonscolombe, 1841	Eastern Palaearctic, Brun and Chazeau (1986)
		***Aphisodinae* (van der Goot, 1917)**	**Oriental and in South Africa**
		*Aphisspiraecola* Patch, 1914	Eastern Palaearctic, Jourdan and Mille (2006)
		***Hysteroneurasetariae* (Thomas, 1878)**	**Nearctic**
		*Rhopalosiphummaidis* (Fitch, 1856)	Central Palaearctic, Brun and Chazeau (1986)
		***Rhopalosiphumnymphaeae* (Linnaeus, 1761)**	**Palaearctic**
		*Rhopalosiphumpadi* (Linnaeus, 1758)	Nearctic, Brun and Chazeau (1986)
		***Rhopalosiphumrufiabdominale* (Sasaki,1899)**	**Eastern Palaearctic, in the Loyalty Islands only**
		***Schizaphisrotundiventris* (Signoret, 1860)**	**Cryptogenic, in the Loyalty Islands only**
	Macrosiphini	***Aulacorthumsolani* (Kaltenbach, 1843)**	**Western Palaearctic**
		***Brachycaudushelichrysi* (Kaltenbach, 1843)**	**Palaearctic**
		*Brevicorynebrassicae* (Linnaeus, 1758)	Palaearctic, Brun and Chazeau (1986)
		*Capitophoruselaeagni* (del Guercio, 1894)	Palaearctic, Brun and Chazeau (1986)
		***Hyperomyzuscarduellinus* (Theobald, 1915)**	**Eastern Palaearctic (Asia), in the Loyalty Islands only**
		*Hyperomyzuslactucae* (Linnaeus, 1758)	Palaearctic, Brun and Chazeau (1986)
		***Lipaphispseudobrassicae* (Davis, 1914)**	**Western Palaearctic**
		*Macrosiphumeuphorbiae* (Thomas, 1878)	Nearctic (North America), Cohic (1958a)
		*Macrosiphumrosae* (Linnaeus, 1758)	Western Palaearctic, Brun and Chazeau (1986)
		***Micromyzuskatoi* (Takahashi, 1925)**	**Oriental**
		***Myzusornatus* Laing, 1932**	**Palaearctic**
		*Myzuspersicae* (Sulzer, 1776)	Eastern Palaearctic, Brun and Chazeau (1986)
		***Pentaloniacaladii* van der Goot, 1917**	**Oriental**
		*Pentalonianigronervosa* Coquerel, 1859	Oriental, first detected in 1991, Jourdan and Mille (2006)
Greenideinae	Greenideini	***Greenideapsidii* van der Goot, 1917**	**Oriental**
Hormaphidinae	Cerataphidini	*Astegopteryxbambusae* (Buckton, 1893)	Oriental, first detected in the sixties, Brun and Chazeau (1986)
		*Cerataphislataniae* (Boisduval, 1867)	Oriental, Brun and Chazeau (1986)
		***Cerataphisorchidearum* (Westwood, 1879)**	**Oriental**
Eriosomatinae	Eriosomatini	***Tetraneurafusiformis* Matsumura, 1917**	**Eastern Palaearctic**

**Table 2.** List of New Caledonian endemic hostplants and their associated aphid species.

Aphid species	Host-plants species (families)
*Aphisaurantii*	*Dodoneaviscosa* (Sapindaceae)
*Aphiseugeniae*	*Glochidionbillardieri* (Myrtaceae)
*Aphisgossypii*	*Hibbertiapancheri* (Dilleniaceae)
	*Myodocarpusfraxinifolius* (Myodocarpaceae)
	*Tarenna* spp. (Rubiaceae)
*Aphisspiraecola*	*Artiabalansae* (Apocynaceae)
	*Ixoracauliflora* (Rubiaceae)
	*Pittosporumcoccineum* (Pittosporaceae)

**Table 3.** Aphid natural enemies in New Caledonia (after Mille 2011; Nattier et al. 2015; Starý 1975).

Orders	Families	Species	Origins	Preys/Hosts
Coleoptera	Coccinellidae	*Apolinuslividigaster* (Mulsant, 1853)	Australasian (only known from Australia, New Zealand and New Caledonia)	An aphid predator specialist
		*Coccinellatransversalis* Fabricius, 1781	Australasian	A polyphagous predator of Aphididae, Psyllidae and Coccoidea
		*Coelophorainaequalis* (Fabricius, 1775)	Australasian	A polyphagous predator of Aphididae and Coccoidea
		*Coelophoramulsanti* (Montrouzier, 1861)	Australasian	A polyphagous predator in natural habitats but also in rangeland and disturbed areas, its preys are psyllids, aphids (*Cerataphis* spp.), and lepidopterous eggs
		*Harmoniaoctomaculata* (Fabricius, 1781) = *Harmoniaarcuata*	Australasian, also known from South Africa	A polyphagous predator of hemiptera including cicadellidae, Aphididae (*Rhopalosiphummaidis*) and Psyllidae
		*Menochilussexmaculatus* (Fabricius, 1781)	Australasian (known from India to Japan and from Western Australia to Lord Howe Island)	Known to prey on *Aphisgossypii* and *Myzuspersicae*
		*Micraspisfrenata* (Erichson, 1842)	Australasian	A hemipterous predator including psyllids, aphids and cicadellids
Diptera	Syrphidae	*Ischiodonscutellaris* (Fabricius, 1805)	Oriental	Numerous species of aphids
Hymenoptera	Braconidae	*Aphidiuscolemani* Viereck, 1912*	Oriental and Australasian	Aphidinae aphids
Neuroptera	Chrysopidae	*Malladabasalis* (Walker, 1853)	Australasian	Numerous species of aphids
	Hemerobiidae	*Eumicromustasmaniae* (Walker, 1860)	Australasian	Numerous species of aphids

* This species was omitted in Mille (2011).

## Discussion

The paucity of native aphids reflects a general property of New Caledonian fauna as already pointed out by previous authors (Zimmerman 1948; Gressitt 1971; Chazeau 1993; Grandcolas et al. 2008), which is a lack of groups that are well represented around the world, and especially with respect to Stenorrhyncha, as already noted (Mille et al. 2016a) for the Coccomorpha. In addition, related predators such as Coccinellidae, especially the coccidivorous and aphidivorous ones also are lacking as native species (Nattier et al. 2015). Native predaceous Coccinellidae are rather specialised in mite predation. The long isolation of New Caledonia can explain such a disharmonic faunal distribution as stated by recent studies (Anso et al. 2016; Nattier et al. 2017).

Comparing the aphid fauna of New Caledonia to that of other analogous island countries (Table 5), the Fiji Islands and Vanuatu have only 13 and 11 species respectively (Sunde et al. 1987; Wilson and Evenhuis 2007), all introduced. In French Polynesia, a list of 23 species was established eleven years ago (Nishida 2008), of which six are significant pest species (Grandgirard 2010). In the Hawaiian Islands, 104 aphid species are present, indicating a much greater influence of commerce compared with South Pacific islands. The situation in New Zealand is quite distinct, with the presence of 12 indigenous recorded aphid species, and a very important introduced fauna of 110 species (Teulon and Stufkens 2002), totalling at least 122 species (Table 5). The larger number of introduced species in New Zealand is probably because the ecology and climate of that country are more similar to that of their areas of origin, than to that of other Pacific or Indian ocean islands. In Réunion Island (Indian Ocean), Remaudière and Etienne (1988) established a list of 45 species, a higher number probably due to the proximity of Africa and Madagascar. Also, ancient and important commercial routes may have played a significant role in the introduction of exotic species in Réunion Island.

It is unique in the regional context that some endemic species are recorded from both Australia (ABRS 2009) and New Zealand (Teulon and Stufkens 1998; Teulon et al. 2010). In New Zealand, two lineages of Aphidina have been found, and Von Dohlen and Teulon (2003) hypothesized that Aphidinae originated in the Southern Hemisphere during the Tertiary and were then able to colonize the Northern Hemisphere, which is controversial if we regard the New Caledonian situation. Conversely, Kim et al. (2011) provided evidence that four endemic Australasian aphidine species originated after divergence from European lineages.

The species most recently discovered in New Caledonia are *Pentaloniacaladii*, *Hyperomyzuscarduellinus*, *Rhopalosiphumrufiabdominale*, and *Schizaphisrotundiventris*, the last three species being found in the Province of the Loyalty Islands, respectively in Tiga, Lifu, and Ouvéa (Figure 1). These new records show the need for a comprehensive survey of aphids within the whole archipelago. From an environmental perspective, a study of aphid impacts on the rich New Caledonian endemic flora should be undertaken in order to evaluate their influence on the ecology of these plants. It is known that aphids cause some environmental issues in Hawai’i for instance as they feed on 64 native Hawaiian plants within 32 botanical families (Mondor et al. 2006; Messing et al. 2007, 2012). It also would be worthwhile to study the influence of aphids on predators and parasitoids, prey and host relationships, and their relationships with other invasive species. However, a related increase of predators (mostly introduced, such as ladybird beetles and lacewings) could jeopardize ecological balances in both agro- and natural ecosystems, although some authors advance the opinion that such environmental impacts are less quantifiable (Teulon and Stufkens 2002). Finally, the presence of these hemipterous insects in the wild can also facilitate the colonization by invasive ants (Le Breton et al. 2005; Idechiil et al. 2007), but could also enhance the spread of beneficial insects from agro-systems. Introduced aphids might disturb existing equilibria between native phytophagous and entomophagous insects. The recent spread of this faunal group may also have been helped by ants, as most invasive ant species are able to tend aphids, resulting in a strong protection for the aphids against predators and parasitoids. The recent arrival of at least 32 exotic ants (Jourdan *in prep.*) during the last century is probably also an important factor promoting the spread of aphids in New Caledonia, as already pointed out for the scale insects (Mille et al. 2016a).

**Figure 2.**
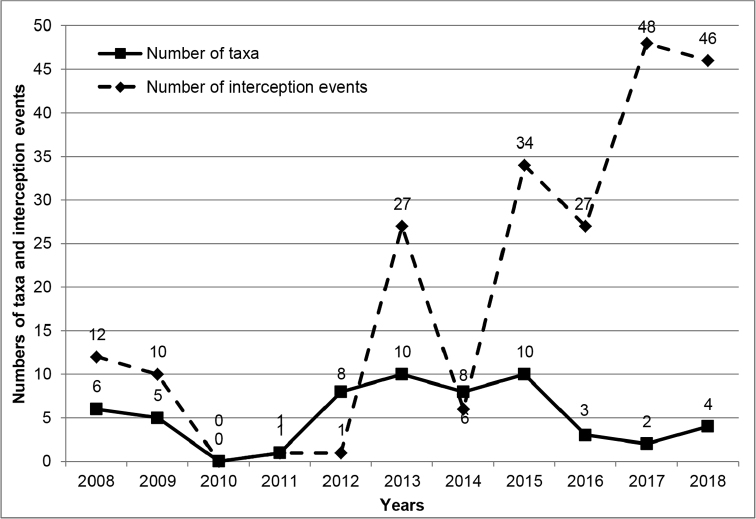
Aphid interceptions in New Caledonia from 2008 to 2018 on fresh imported fruits and vegetables.

With 33 exotic species introduced during a period of 165 years (1853–2018, counting from the incorporation of the archipelago in France in 1853 to the present), the average rate of introduction is 0.20 species per year. In comparison, in the Hawaiian archipelago (discovered in 1778), 105 species of Aphidoidea (incl. one species of Adelgidae) have become established with an average rate of introduction of 0.82 species per year –four times the rate in New Caledonia– from 1910 to 2012 (Foottit et al. 2012). Like New Caledonia, Hawai’i does not have any native aphid species (Foottit et al. 2012). The closeness of the climates of these two archipelagos shows that New Caledonia potentially could host many other species of aphids. New Zealand has an introduced fauna of 110 species, but differs in that there are more than a dozen endemic species (Teulon and Stufkens 2002). There, the rate of introduction is estimated at 0.85 aphid per year. The low rate of introduction for New Caledonia can be explained mainly because the archipelago was not on major commercial routes until recently. In the last decade the number of interception events in New Caledonia has greatly increased (Figure 2, Table 4). In New Zealand, the rate of introduction of alien aphid species has declined dramatically in recent years (Teulon and Stufkens 2002), probably because of the strong biosecurity policy and efforts that are deployed at ports of entry to New Zealand, as also observed earlier in North America during the thirties (Skvarla et al. 2017). Increased biosecurity scrutiny is obviously a major tool to prevent the spread of these economically important pests.

**Table 4.** List of the aphid species intercepted by the Biosecurity Services (DAVAR-SIVAP) in New Caledonia but still considered unestablished.

**Subfamilies**	**Tribes**	**Species / Common names / Noms communs**	**Intercepted commodity**	**Country of origin**	**Biogeographic region of origin**
Aphidinae	Aphidini	***Aphis* sp.**	Parsley (*Petroselinumcrispum*, Apiaceae)	Australia	–
	Macrosiphini	***Acyrthosiphonlactucae* (Passerini, 1860)**	Lettuce (*Lactucasativa*, Asteraceae)	Australia	Palaearctic
		***Cavariellaaegopodii* (Scopoli, 1763)**	Parsley (*Petroselinumcrispum*, Apiaceae)	Australia	Western Palaearctic
		Willow-carrot Aphid / Puceron de la Carotte			
		***Chaetosiphonfragaefolii* (Cockerell, 1901)**	Strawberry (*Fragaria* spp., Rosaceae)	USA	Nearctic (North America)
		Strawberry Aphid / Puceron jaune du Fraisier			
		***Dysaphisapiifolia* (Theobald, 1923)**	Fennel (*Foeniculumvulgare*, Apiaceae)	New Zealand	Palaearctic
		***Dysaphisfoeniculi* (Passerini, 1860)**	Fennel (*Foeniculumvulgare*, Apiaceae)	Australia	Western Palaearctic
		***Dysaphislappae* (Koch, 1854)** Thistle Root Aphid	Artichoke (*Cynarascolymus*, Asteraceae)	Australia	Palaearctic
		***Hyadaphiscoriandri* (Das, 1918)**	Fennel (*Foeniculumvulgare*, Apiaceae)	New Zealand	Palaearctic
		Coriander Aphid / Puceron de la Coriandre			
		***Hyadaphispasserini* (Del Guercio, 1911)**	Fennel (*Foeniculumvulgare*, Apiaceae)	New Zealand	Palaearctic
		Honeysuckle Aphid / Puceron du Chèvrefeuille			
		***Myzusascalonicus* Doncaster, 1946**	Fennel (*Foeniculumvulgare*, Apiaceae) and Celery (*Apiumgraveolens*, Apiaceae but this species is highly polyphagous	Australia	Unknonwn
		Shallot aphid / Puceron de l’Échalote			
		***Nasonoviaribisnigri* (Mosley, 1841)**	Lettuce (*Lactucasativa*, Asteraceae)	New Zealand	Western Palaearctic
		Lettuce Aphid / Puceron de la Laitue			
Eriosomatinae		***Eriosomalanigerum* (Hausmann, 1802)**	Apple (*Maluspumila*, Rosaceae)	France	Palaearctic?
		Woolly Apple Aphid / Puceron lanigère du Pommier			
Lachninae	Eulachnini	***Cinaratujafilina* (Del Guercio, 1909)**	On two cypress trees	Unknown	Palaearctic
		Cypress Pine Aphid / Puceron du Thuya			

**Figure 3.**
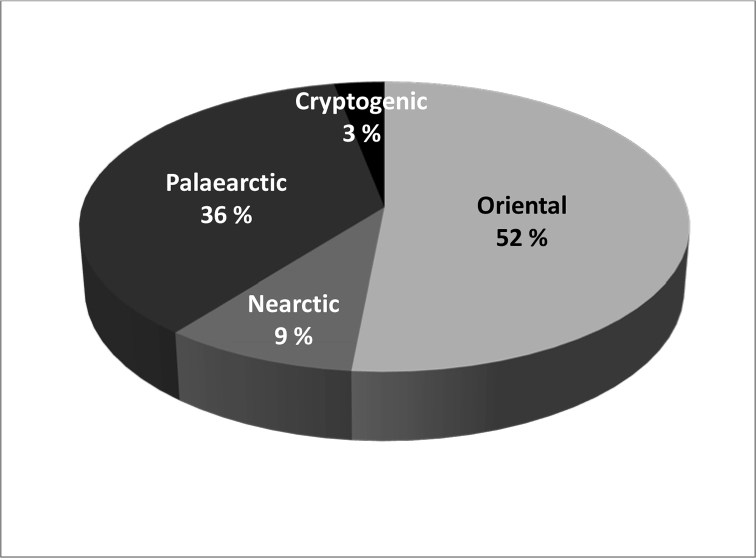
Biogeographic origin of the 33 aphid species present in New Caledonia.

The biogeographic origins of introduced aphid species in New Caledonia are mainly distributed between Oriental (52%, incl. Eastern Palaearctic) and Palaearctic (36%), only 9% being of Nearctic origin, plus one cryptogenic species (*Schizaphisrotundiventris*) (Figure 3). This compares with Hawai’i, where introduced aphids are 35% Oriental (incl. Eastern Palaearctic), 35% West Palaearctic, and 21% Nearctic (Foottit et al. 2012). One can assume that different patterns of trade affect the probability that species from certain biogeographic regions are introduced. However, the low rate of establishment in New Caledonia might also be partly explained by climatic mismatching between the countries involved. Biogeographic connections may also help to explain the low numbers of introduced aphid species in more tropical islands such as Fiji Islands, French Polynesia, and Vanuatu, although the pattern of trade may also differ according to lifestyle (lower or no import of fresh commodities such as vegetables or fruits).

The recent increase in imports of fresh commodities from two large neighboring countries (Australia and New Zealand) increases the risk of accidental introductions of new species. This is illustrated by Figure 2 and Table 4 showing the increase of interception events and intercepted specimens from 2008 to the present, particularly from these two countries. Table 4 shows that 80% of the regularly intercepted species are originally from the Palaearctic and the remaining 20% are from the Nearctic, most of them being in the tribe Macrosiphini. Some of the intercepted species originating from the Northern Hemisphere may not be able to adapt to the New Caledonian environments, but several examples show that some Northern Hemisphere aphids can adapt to New Caledonia biotopes. Eighty percent of aphids originating from Palaearctic or Western Palaearctic regions seem unfitted to colonise New Caledonia (Table 4), but repeated incursions may lead to introduction of more adapted strains, as we already have observed settlement of some Palaearctic species in the archipelago (Figure 3). This applies especially to species such as *Nasonoviaribisnigri*, which has been repeatedly intercepted in imports from New Zealand to New Caledonia since at least 2008. One can note the recent interceptions of *Dysaphisapiifolia* from Australia and New Zealand in 2017 and 2018, *Myzusascalonicus* from Australia and New Zealand in March, June, and October 2019, and *Eriosomalanigerum* from France in February 2018. In 2015, the Strawberry Aphid, *Chaetosiphonfragaefolii* was intercepted on strawberries imported from the USA. Establishment of *C.fragaefolii* in New Caledonia would bring a new pest in this crop, already attacked by many other pests and diseases. At the moment, these 13 species are not established in New Caledonia, but their recurrent interceptions might result in a future settlement, especially in the case of *Myzusascalonicus*, because it is highly polyphagous and is known on potatoes which are cultivated in New Caledonia. Obviously, continued and enhanced surveillance of imported commodities is needed. Finally, *Cinaratujafilina* was discovered on the 12th October 2018 on two cypress trees in a garden of Nouméa. An eradication program was subsequently launched by the Biosecurity Services (DAVAR-SIVAP). This species can be considered as a potential and significant threat to endemic and endangered species of Cupressaceae, especially species of the genera *Callitris* and *Libocedrus*.

**Table 5.** Comparison of aphid fauna (excl. Adelgidae and Phylloxeridae) between seven island countries (after Wilson and Evenhuis (2007) for Fiji Islands, Nishida (2008) and Grandgirard (2010) for French Polynesia, Foottit et al. (2012) and Messing et al. (2012) for Hawai’i, Macfarlane et al. (2010), Teulon et al. (2010, 2013) for New Zealand, Remaudière and Etienne (1988) for Réunion Island, Sunde et al. (1987) for Vanuatu).

	Island countries						
Aphid subfamilies	Fiji Islands	French Polynesia	Hawaii (USA)	New Caledonia	New Zealand	Réunion Island	Vanuatu
Aphidinae	11	20	80	28	78	38	8
Calaphidinae	0	0	3	0	13	0	0
Chaitophorinae	0	0	2	0	3	0	0
Eriosomatinae	1	0	4	1	10	2	0
Greenideinae	0	0	1	1	0	0	0
Hormaphidinae	1	3	5	3	2	3	3
Lachninae	0	0	7	0	7	2	0
Phyllaphidinae	0	0	0	0	1	0	0
Neophyllaphidinae	0	0	2	0	2	0	0
Saltusaphidinae	0	0	0	0	1	0	0
Taiwanaphidinae	0	0	0	0	1	0	0
**Total taxa**	**13**	**23**	**104**	**33**	>122*	**45**	**11**

* This approximative number does not comprise the four species of Adelgidae, the three of Phylloxeridae, and the 18 native species, making a total of 154 species of Aphidoidea for New Zealand (David Teulon, pers. comm. 19 July 2018).

## Conclusions

To our present knowledge, no aphids occurred in New Caledonia before European settlements. The present updated species list is an important step to better secure the trade in fresh commodities. It is imperative to set up some strict regulations concerning the movement of fresh commodities, especially from the countries where the regularly intercepted species are present. In New Zealand, Teulon and Stufkens (2002) reminded us that “Aspects of aphid biology, such as small size, parthenogenetic reproduction, high reproductive rates, short generation time, rapid dispersal and eruptive population dynamics, pose particularly difficult challenges for aphid biosecurity in New Zealand”. This statement also is highly relevant for a subtropical “biodiversity-hotspot” country such as New Caledonia, where there are no endemic aphids.

Apart from virus transmissions (chiefly BBTV and CTV), direct damage by aphids does not constitute a major problem in New Caledonian orchards, probably because of the significant activities of predators and parasitoids. However, damage due to virus transmission in field crops, especially in squash (*Cucurbitapepo*) and several other crops, can be economically significant (Bordat and Daly 1995).

All 33 species appear to have been introduced accidentally by human activity in the last 100 years. Thirteen more species also are intercepted more or less regularly at the borders through biosecurity surveys, without further establishment. This demonstrates that aphids represent a major biosecurity threat, including the one as potential plant virus vectors. Consequently, the reinforcement of biosecurity is a priority for such biodiversity hotspots, from both the perspective of agriculture and of the native environment. Of course, these measures cannot guard against the long-distance dispersal of such low-weight insects as aphids on air currents, as stated by some authors (Johnson 1967). Even some heavier insects are already known to fly over several hundred to thousand kilometers over the sea, with *Calligraphapantherina* Stål (Coleoptera, Chrysomelidae) as a recent example for New Caledonia (Mille et al. 2016b).

Furthermore, prioritization and promotion of local development of vegetable and fruit crops, rather than their risky importation from abroad, is desirable. Such an approach also should be promoted and extended to other Pacific islands which all share the lack of native aphid fauna and associated plant virus vector risks. Also, as a consequence of global climate change, the regularly intercepted species could find their ecological requirements, settle and dramatically change the fragile ecological balance in this insular biodiversity hotspot. There is an urgent need for a plant quarantine facility in New Caledonia (Cohic 1958b; Mille et al. 2016a), accompanied by some strict regulations against these and other quarantined insects.

## Dedication

We dedicate this article to the late Professor François Leclant (22 July 1934–14 January 2001), INRA, Montpellier, France, who trained one of us (CM) in the study of aphids, and more widely, in Agricultural Entomology, and to the late Mrs. Rosa C. Henderson (1 June 1942–13 December 2012) who trained one of us (SC) in the preparation of slides of aphids, other soft insects, and mites. We openly thank her, who encouraged two of us (SC and CM) to write the present article about aphids of New Caledonia.

## References

[B1] ABRS (2009) Australian Faunal Directory. Aphididae. Australian Biological Resources Study, Canberra. http://www.environment.gov.au/biodiversity/abrs/online-resources/fauna/afd/taxa/APHIDIDAE [accessed on 22 March 2013]

[B2] Agarwala BK Ghosh AK (1988) Prey records of Aphidophagous coccinellids of India. A review and bibliography. Tropical Pest Management 34(1): 1–14. https://doi.org/10.1080/09670878809371196

[B3] Anonymous (2010) Inventaire 2010 du Verger Calédonien et Évaluation de la Consommation de Fruits Frais. Service de l’eau et des statistiques et études rurales (SESER), Affaires rurales, section statistiques, Juillet 2010, 38 pp.

[B4] Anso J Barrabé L Desutter-Grandcolas L Jourdan H Grandcolas P Dong J Robillard T (2016) Old Lineage on an Old Island: *Pixibinthus*, a New Cricket Genus Endemic to New Caledonia Shed Light on Gryllid Diversification in a Hotspot of Biodiversity. PLOS ONE 11(3): e0150920. https://doi.org/10.1371/journal.pone.0150920 10.1371/journal.pone.0150920 PMC4814057 27027632

[B5] Batabyal AA Beladi H (2006) International trade and biological invasions: A queuing theoretic analysis of the prevention problem. European Journal of Operational Research 170: 758–770. https://doi.org/10.1016/j.ejor.2004.07.065

[B6] Bayer RJ Mabberley DJ Morton C Miller CH Sharma IK Pfeil BE Rich S Hitchcock R Sykes S (2009) A molecular phylogeny of the orange subfamily (Rutaceae: Aurantioideae) using nine cpDNA sequences. American Journal of Botany 96(3): 668–685. https://doi.org/10.3732/ajb.0800341 21628223 10.3732/ajb.0800341

[B7] Beardsley JW (1993) *Greenideaformosana* (Maki), an aphid new to the Hawaiian Islands (Homoptera: Aphididae: Greenideinae). Proceedings of the Hawaiian Entomological Society 32: 157–158.

[B8] Beauvais M-L Coléno A Jourdan H (2006) Les Espèces Envahissantes Dans L’archipel Néo-Calédonien. Un risque environnemental et économique majeur. IRD Éditions, Collection Expertise collégiale, Paris, 260 pp. https://doi.org/10.4000/books.irdeditions.7612

[B9] Blackman RL Eastop VF (1984) Aphids on the World’s Crops – Identification and Information Guide. John Wiley Sons, London, 476 pp.

[B10] Blackman RL Eastop VF (1994) Aphids on the World’s Trees. CAB International in association with The Natural History Museum, Wallingford, 987 pp.

[B11] Blackman RL Eastop VF (2000) Aphids on the World’s Crops – An Identification and Information Guide (2^nd^ edn.). John Wiley & Sons, Chichester, 414 pp.

[B12] Blackman RL Eastop VF (2006) Aphids on the World’s Herbaceous Plants and Shrubs (2 volume set). John Wiley & Sons, Chichester, 1460 pp.

[B13] Blackman RL Eastop VF (2007) Taxonomic issues, 1–29. In: van Emden HF, Harrington R (Eds) Aphids as Crop Pests. CAB International, Wallingford. https://doi.org/10.1079/9780851998190.0001

[B14] Blackman RL Eastop VF (2020) Aphids on the World’s Plants. An Identification and Information Guide. http://www.aphidsonworldsplants.info/ [accessed on 7 February 2020]

[B15] Blackman RL Sorin M Miyazaki M (2011) Sexual morphs and colour variants of *Aphis* (formerly *Toxoptera*) *odinae* (Hemiptera, Aphididae) in Japan. Zootaxa 3110: 53–60. https://doi.org/10.11646/zootaxa.3110.1.5

[B16] Boakye DB Randles JW (1974) Epidemiology of Lettuce necrotic yellow virus in south Australia. III: Virus transmission parameters and vector feeding behaviour in host and non host plants. Australian Journal of Agricultural Research 25: 791–802. https://doi.org/10.1071/AR9740791

[B17] Bordat D Daly P (1995) Catalogue des Principaux Arthropodes Présents sur les Cultures Légumières de Nouvelle-Calédonie. CIRAD-FLHOR, CIRAD/Mandat de gestion de Nouvelle-Calédonie, 95 pp.

[B18] Brun L-O Chazeau J (1986) Catalogue des ravageurs d’intérêt agricole de Nouvelle-Calédonie (2^ième^ edn). ORSTOM, Centre de Nouméa, 130 pp.

[B19] CABI (2018) *Aphiscraccivora*. Angela Whittaker. Invasive Species Compendium. CAB International, Wallingford. https://www.cabi.org/isc/datasheet/6192 [accessed on 6 February 2018]

[B20] CABI (2019) *Toxopteraaurantii* (camellia aphid). Invasive Species Compendium. CAB International, Wallingford. https://www.cabi.org/isc/datasheet/54270 [accessed on 7 October 2019]

[B21] Cambra M Gorris MT Marroquin C Roman MP Olmos A Martinez MC Hermoso De Mendoza A Lopez A Navarro L (2000) Incidence and epidemiology of *Citrustristeza virus* in the Valencian Community of Spain. Virus Research 71: 85–95. https://doi.org/10.1016/S0168-1702(00)00190-8 11137164 10.1016/s0168-1702(00)00190-8

[B22] Chan CK Forbes AR Raworth DA (1991) Aphid-Transmitted Viruses and Their Vectors of the World. Agriculture Canada Technical Bulletin 1991-3E, 216 pp.

[B23] Chen C Weng CH (1969) Studies on species and occurrence of winged aphids on tobacco. Plant Protection Bulletin, Taiwan 11(2): 71–76.

[B24] Charles JG Henderson RC (2002) Catalogue of the exotic armoured scale insects (Hemiptera: Coccoidea: Diaspididae) in New Zealand. Journal of the Royal Society of New Zealand 32(4): 587–615. https://doi.org/10.1080/03014223.2002.9517711

[B25] Chazeau J (1993) Research on New Caledonian terrestrial fauna: achievements and prospects. Biodiversity Letters 1: 123–129. https://doi.org/10.2307/2999756

[B26] Coeur d’acier A Pérez Hidalgo N Petrović-Obradović O (2010) Aphids (Hemiptera, Aphididae). Chapter 9.2. In: Roques A Kenis M Lees D Lopez-Vaamonde C Rabitsch W Rasplus J-Y Roy D (Eds) Terrestrial alien arthropods of Europe.BioRisk 4(1), 435–474. https://doi.org/10.3897/biorisk.4.57

[B27] Cohic F (1956) Parasites Animaux des Plantes Cultivées en Nouvelle-Calédonie et Dépendances. Office de la Recherche Scientifique et Technique Outre-Mer, Institut Français d’Océanie, Nouméa, 94 pp.

[B28] Cohic F (1958a) Les Parasites Animaux de la Tomate en Nouvelle-Calédonie. Office de la Recherche Scientifique et Technique Outre-Mer, Institut Français d’Océanie, Nouméa, 9 pp.

[B29] Cohic F (1958b) Contribution à L’étude des Cochenilles D’intérêt Économique de Nouvelle-Calédonie et Dépendances. South Pacific Commission, Technical Paper 116, 35 pp.

[B30] Culik MP Ventura JA Martins D Dos S (2016) Range expansion of the invasive insect Greenidea (Trichosiphon) psidii (Hemiptera: Aphididae) in the neotropical region. Springerplus 5(1): 1–734. https://doi.org/10.1186/s40064-016-2487-8 27376002 10.1186/s40064-016-2487-8 PMC4909684

[B31] D’Arcy CJ Burnett PA Hewings AD (1981) Detection, biological effects, and transmission of a virus of the aphid *Rhopalosiphumpadi*. Virology 114: 268–272. https://doi.org/10.1016/0042-6822(81)90275-0 18635095 10.1016/0042-6822(81)90275-0

[B32] Dawson W Moser D van Kleunen M Kreft H Pergl J Pyšek P Weigelt P Winter M Lenzner B Blackburn TM Dyer EE Cassey P Scrivens SL Economo EP Guénard B Capinha C Seebens H García-Díaz P Nentwig W García-Berthou E Casal C Mandrak NE Fuller P Meyer C Essl F (2017) Global hotspots and correlates of alien species richness across taxonomic groups. Nature, ecology and evolution 1: 0186. https://doi.org/10.1038/s41559-017-0186

[B33] Eastop VF (1966) A taxonomic study of Australian Aphidoidea (Homoptera). Australian Journal of Zoology 14(3): 399–592. https://doi.org/10.1071/ZO9660399

[B34] Elliott NC French BW Burd JD Kindler SD Reed DK (1994) Parasitism, adult emergence, sex ratio, and size of *Aphidiuscolemani* (Hymenoptera: Aphidiidae) on several aphid species. The Great Lakes Entomologist 27(3): 137–142. http://scholar.valpo.edu/tgle/vol27/iss3/2

[B35] Etzel RW Petitt FL (1992) Scientific Notes: Association of *Verticilliumlecanii* with Population Reduction of Red Rice Root Aphid (*Rhopalosiphumrufiabdominalis*) on Aeroponically Grown Squash. Florida Entomologist 75(4): 605–606. https://doi.org/10.2307/3496144

[B36] Favret C (2018) Aphid species file. Version 5.0/5.0.[2018.01]. http://Aphid.SpeciesFile.org [accessed on 5 January 2018]

[B37] Foottit RG Maw HEL Pike KS Miller RH (2010) The identity of *Pentalonianigronervosa* Coquerel and *P.caladii* van der Goot (Hemiptera: Aphididae) based on molecular and morphometric analysis. Zootaxa 2358: 25–38. https://doi.org/10.11646/zootaxa.2358.1.2

[B38] Foottit RG Maw HEL Pike KS Messing RH (2012) The aphids (Hemiptera: Aphididae and Adelgidae) of Hawai’i: Annotated list and key to species of an adventive fauna. Pacific Science 66(1): 1–30. https://doi.org/10.2984/66.1.1

[B39] Fournier V Brodeur J (1999) Biological control of lettuce aphids with the entomopathogenic fungus *Verticilliumlecanii* in greenhouses. IOBC/WPRS Working Group “Integrated Control in Glasshouses”. Proceedings of the meeting at Brest, France 25–29 May 1999, 77–80.

[B40] Ghorpadé KD (1981) Insect prey of Syrphidae (Diptera) from India and neighbouring countries: A Review and Bibliography. Tropical Pest Management 27: 62–82. https://doi.org/10.1080/09670878109414173

[B41] Grandcolas P Murienne J Robillard T Desutter-Grandcolas L Jourdan H Guilbert E Deharveng L (2008) New Caledonia: a very old Darwinian island? Philosophical Transactions of the Royal Society B 363: 3309–3317. https://doi.org/10.1098/rstb.2008.0122 10.1098/rstb.2008.0122 PMC2607381 18765357

[B42] Grandgirard J (2010) Guide de Reconnaissance des Insectes et Acariens des Cultures Maraîchères, Fruitières et Vivrières de Polynésie Française. Service du Développement Rural, 147 pp.

[B43] Gressitt JL (1971) Relative faunal disharmony of insects on Pacific Islands. Entomological Essays to Commemorate the Retirement of Professor K. Yasumatsu. Hokuryukan Publishing Co., Ltd., Tokyo, 15–24.

[B44] Hadi BAR Flanders KL Bowen KI Murphy JF Halbert SE (2011) Species composition of aphid vectors (Hemiptera: Aphididae) of Barley yellow dwarf virus and Cereal yellow dwarf virus in Alabama and western Florida. Journal of Economic Entomology 104(4): 1167–1173. https://doi.org/10.1603/EC10425 21882679 10.1603/ec10425

[B45] Halbert SE Brown LG (1998) *Toxopteracitricida* (Kirkaldy), Brown Citrus Aphid – Identification, Biology, and Management Strategies. Entomology Circular N° 374, Florida Department of Agriculture & Consumer Services, Division of Plant Industry, 6 pp.

[B46] Hopkinson JE Kramer SM Zalucki MP (2016) Developmental biology and prey preference of *Diomusnotescens* Blackburn (Coleoptera: Coccinellidae): A predator of *Aphisgossypii* Glover (Hemiptera: Aphididae). Biological Control 96: 101–107. https://doi.org/10.1016/j.biocontrol.2016.02.006

[B47] Hull FM (1937) A Check List of the Syrphidae of Oceania. Occasional Papers of Bernice P. Bishop Museum, Honolulu, Hawaii 13(10): 79–87.

[B48] Hullé M Turpeau E Chaubet B (2006) Encyclop’ahid. INRA. https://doi.org/10.15454/1.4333379890530916E12

[B49] Hulme PE (2009) Trade, transport and trouble: managing invasive species pathways in an era of globalization. Journal of Applied Ecology 46: 10–18. https://doi.org/10.1111/j.1365-2664.2008.01600.x

[B50] Idechiil O Miller RH Pike KS Hansen LD (2007) Aphids (Hemiptera: Aphididae), ants (Hymenoptera: Formicidae) and associated flora of Palau with comparisons to other Pacific Islands. Micronesica 39(2): 141–170.

[B51] Johnson CG (1967) International dispersal of insects and insect-borne viruses. Netherlands Journal of Plant Pathology 73: 21–43. https://doi.org/10.1007/BF01974421

[B52] Joshi S Ballal CR (2013) Syrphid predators for biological control of aphids. Journal of Biological Control 27(3): 151–170.

[B53] Jouda G Monia BHK Naima B (2010) First report of aphidopathogenic fungi *Fusariumsemitectum* (Berkeley and Ravenel, 1875) and *Fusariumsacchari* (Butler and Hafiz Khan) Gams (1971) on *Capitophoruselaeagni* (Del Guercio) (Hemiptera: Aphididae). African Journal of Agricultural Research 5(4): 290–293.

[B54] Jourdan H Mille C (2006) Les invertébrés introduits dans l’archipel néo-Calédonien: espèces envahissantes et potentiellement envahissantes. Première Évaluation et Recommandation Pour leur Gestion. Expertise Collégiale IRD, Paris, 168–219.

[B55] Kagy V Thomas JE Sharman M Mademba-Sy F (2001) First record of banana bunchy top disease in New Caledonia. Australasian Plant Pathology Society 30(1): 1–71. https://doi.org/10.1071/AP00065

[B56] Kalaitzaki A Awad S Malandraki E Papapetrou PD Livieratos I Margaritopoulos JT (2019) Aphid species composition in populations from citrus orchards in a region of the island of Crete. Bulletin of Insectology 72(1): 143–149.

[B57] Kavallieratos NG Stathas GJ Tomanovic Z (2004) Seasonal abundance of parasitoids (Hymenoptera: Braconidae, Aphidiinae) and predators (Coleoptera: Coccinellidae) of aphids infesting citrus in Greece. Biologia (Bratislava) 59(2): 191–196.

[B58] Kennedy JS Day MF Eastop VF (1962) A Conspectus of Aphids as Vectors of Plant Viruses. Commonwealth Institute of Entomology, London, 114 pp.

[B59] Kim H Lee S Jang Y (2011) Macroevolutionary patterns in the Aphidini aphids (Hemiptera: Aphididae): Diversification, host association, and biogeographic origins. PLoS ONE 6(9): e24749. https://doi.org/10.1371/journal.pone.0024749 10.1371/journal.pone.0024749 PMC3174202 21935453

[B60] Le Breton J Jourdan H Chazeau J Orivel J Dejean A (2005) Niche opportunity and ant invasion: The case of *Wasmanniaauropunctata* in a New Caledonian rain forest. Journal of Tropical Ecology 21: 93–98. https://doi.org/10.1017/S0266467404002019

[B61] Lee RF Keremane ML (2013) Mild strain cross protection of tristeza: A review of research to protect against decline on sour orange in Florida. Frontiers in Microbiology 4: 1–259. https://doi.org/10.3389/fmicb.2013.00259 24046764 10.3389/fmicb.2013.00259 PMC3764332

[B62] Lohr C Wenger A Woodberry O Pressey RL Morris K (2017) Predicting island biosecurity risk from introduced fauna using Bayesian Belief Networks. Science of The Total Environment 601 (Supplement C): 1173–1181. https://doi.org/10.1016/j.scitotenv.2017.05.281 10.1016/j.scitotenv.2017.05.281 28605835

[B63] Lopez T Libert P-N Starý P Japoshvili G Hatt S Francis F (2016) Checklist of Aphidiinae (Hymenoptera: Braconidae) and *Aphelinus* (Hymenoptera: Aphelinidae) species from Belgium with respectively four and three new records. Zootaxa 4092(4): 548–560. https://doi.org/10.11646/zootaxa.4092.4.5 27394473 10.11646/zootaxa.4092.4.5

[B64] Mackauer M (1968) Insect parasites of the green peach aphid, *Myzuspersicae* Sulz., and their control potential. Entomophaga 13: 91–106. https://doi.org/10.1007/BF02371780

[B65] Macfarlane RP Maddison PA Andrew IG Berry JA Johns PM Hoare RJB Larivière M-C Greenslade P Henderson RC Smithers CN Palma RL Ward JB Pilgrim RLC Towns DR McLellan I Teulon DAJ Hitchings TR Eastop VF Martin NA Fletcher MJ Stufkens Dale PJ Burckhardt D Buckley TR Trewick SA (2010) Phylum Arthropoda subphylum Hexapoda: Protura, springtails, Diplura, and insects. In: Gordon D (Ed.) New Zealand Inventory of Biodiversity (Vol.2). Canterbury University Press, Christchurch, 233–467.

[B66] Manfrino RG Zumoffen L Salto CE Lopez Lastra CC (2013) Potential plant-aphid-fungal associations aiding conservation biological control of cereal aphids in Argentina. International Journal of Pest Management 59(4): 314–318. https://doi.org/10.1080/09670874.2013.869372

[B67] McAuslane HJ (2017) Oleander aphid, *Aphisnerii* Boyer de Fonscolombe (Insecta: Hemiptera: Aphididae). EENY-247. http://entnemdept.ufl.edu/creatures/orn/shrubs/oleander_aphid.htm

[B68] Messing RH Rabasse JM (1995) Oviposition behaviour of the polyphagous aphid parasitoid *Aphidiuscolemani* Viereck (Hymenoptera: Aphidiidae). Agriculture, Ecosystems and Environment 52: 13–17. https://doi.org/10.1016/0167-8809(94)09002-O

[B69] Messing RH Tremblay MN Mondor EB Foottit RG Pike KS (2007) Invasive aphids attack native Hawaiian plants. Biological Invasion 9: 601–607. https://doi.org/10.1007/s10530-006-9045-1

[B70] Messing RH Pike KS Foottit RG (2012) Invasive Aphids in Hawaii. College of Tropical Agriculture and Human Resources, University of Hawai’i at Mánoa, 261 pp.

[B71] Mille C (2000) Le Puceron du Bananier, *Pentalonianigronervosa* Coquerel (Homoptera : Aphididae), l’insecte vecteur de la Maladie virale du Bunchy top. I.A.C., Programme Cultures Fruitières, SRFP, Protection phytosanitaire des cultures fruitières en Nouvelle-Calédonie, Fiche technique 3.5, 3 pp.

[B72] Mille C (2011) Animaux Nuisibles et Utiles des Jardins et Vergers de Nouvelle-Calédonie. Éditions SENC, Société Entomologique de Nouvelle-Calédonie, 200 pp.

[B73] Mille C Cazères S (2018) Rapport technique et financier 2017. Convention relative au dispositif d’identification d’espèces exogènes en Nouvelle-Calédonie pour l’année 2017 entre le SIVAP, le GDS-V et l’IAC, 22 pp.

[B74] Mille C Henderson RC Cazères S Jourdan H (2016a) Checklist of the scale insects (Hemiptera: Sternorrhyncha: Coccomorpha) of New Caledonia. Zoosystema 38(2): 129–176. https://doi.org/10.5252/z2016n2a1

[B75] Mille C Rigault F Cazères S Jourdan H (2016b) Recent spread in New Caledonia of the Sida leafbeetle, *Calligraphapantherina* Stål, 1859 (Coleoptera: Chrysomelidae: Chrysomelinae). Check List 12(1): 1–5. https://doi.org/10.15560/12.1.1837

[B76] Mondor EB Tremblay MN Messing RH (2006) Morphological and ecological traits promoting aphid colonization of the Hawaiian Islands. Biological Invasion 9(1): 87–100. https://doi.org/10.1007/s10530-006-9010-z

[B77] Montrouzier X (1861) Essai sur la faune entomologique de la Nouvelle-Calédonie (Balade) et des îles des Pins, Art, Lifu, etc. HÉMIPTÈRES (1). Annales de la Société entomologique de France 4(1): 59–74.

[B78] Nasruddin A (2013) First record of *Hysteroneurasetariae* (Hemiptera: Aphididae) on rice in South Sulawesi, Province of Indonesia. Florida Entomologist 96(2): 647–648. https://doi.org/10.1653/024.096.0237

[B79] Nattier R Jourdan H Mille C Chazeau J (2015) Annotated checklist and distribution of the ladybird beetles (Coleoptera: Coccinellidae) from New Caledonia. Zootaxa 4058(3): 301–331. https://doi.org/10.11646/zootaxa.4058.3.1 26701529 10.11646/zootaxa.4058.3.1

[B80] Nattier R Pellens R Robillard T Jourdan H Legendre F Caesar M Nel A Grandcolas P (2017) Updating the phylogenetic dating of New Caledonian biodiversity with a meta-analysis of the available evidence. Nature Scientific Reports 7: 1–3705. https://doi.org/10.1038/s41598-017-02964-x 10.1038/s41598-017-02964-x PMC5473893 28623347

[B81] Naumann-Etienne K Remaudière G (1995) A commented preliminary checklist of the aphids (Homoptera: Aphididae) of Pakistan and their host plants. Parasitica 51(1): 3–61.

[B82] Nishida GM (2008) French Polynesia Bug Checklist (Preliminary). Version November 13, 2008. http://essigdb.berkeley.edu/checklists/ [accessed on 4 August 2015]

[B83] O’Connor BA (1969) Exotic Plant Pests and Diseases – Ennemis et Maladies Exotiques des Végétaux. South Pacific Commission, Nouméa, 460 pp.

[B84] Oraze MJ Grigarick AA (1992) Biological control of duck salad (*Heterantheralimosa*) by the waterlily aphid (*Rhopalosiphumnymphaeae*) in rice (*Oryzasativa*). Weed Science 40: 333–336. https://doi.org/10.1017/S004317450005743X

[B85] Paliwal YC (1980) Transmission of barley yellow dwarf isolates by the cereal root aphid *Rhopalosiphumrufiabdominalis*. Canadian Journal of Plant Pathology 2(2): 90–92. https://doi.org/10.1080/07060668009501445

[B86] Péréfarres F De Bruyn A Kraberger S Hoareau M Barjon F Lefeuvre P Pellegrin F Caplong P Varsani A Lett J-M (2012) Occurrence of the Israel strain of *Tomato yellow leaf curl virus* in New Caledonia and Loyalty Islands. New Disease Reports 25: 1–6. https://doi.org/10.5197/j.2044-0588.2012.025.006

[B87] Pérez Hidalgo N González Hernández A Carnero Hernández A Seco Fernández MV (2000) Dos especies de *Cerataphis* (Hemiptera, Aphididae: Hormaphidinae) introducidas en las Islas Canarias. Boletín de Sanidad Vegetal Plagas 26: 425–432.

[B88] Rakauskas R Bašilova J Bernotienė R (2015) *Rhopalosiphumrufiabdominale*: First records from winter host plants in Europe. Bulletin of Insectology 68: 73–81.

[B89] Rattanapun W (2017) Banker plant system using *Hysteroneurasetariae* (Thomas) (Hemiptera: Aphididae) as a non-pest prey to build up the lady beetle populations. Journal of Asia-Pacific Entomology 20: 437–440. https://doi.org/10.1016/j.aspen.2017.02.016

[B90] Remaudière G Etienne J (1988) Les Aphididae (Hom.) des îles et archipels de l’Océan Indien. L’AgronomieTropicale 43(4): 327–346.

[B91] Remaudière G Remaudière M (1997) Catalogue des Aphididae du Monde, HomopteraAphidoidea. INRA Editions, 478 pp.

[B92] Roy S Rahman A (2014) A study on the comparative predatory efficiency and development of *Micraspisdiscolor* (F.) and *Menochilussexmaculatus* (F.) on tea aphid *Toxopteraaurantii* (Boyer de Fons.). Zoology and Ecology 24(3): 285–287. https://doi.org/10.1080/21658005.2014.933632

[B93] Sallée B Chazeau J (1985) Cycle de développement, table de vie, et taux intrinsèque d’accroissement en conditions contrôlées de *Coelophoramulsanti* (Montrouzier), Coccinellidae aphidiphage de Nouvelle-Calédonie (Coleoptera). Annales de la Société entomologique de France 21: 407–412.

[B94] Sarma KK Dutta SK Borah BK (1996) Interaction between *Aphiscraccivora* Koch and its predators, *Coccinellatransversalis* Fabricius (Coleoptera: Coccinellidae) and *Ischiodonscutellaris* (Fabricius) (Diptera: Syrphidae). Journal of Biological Control 10: 125–128.

[B95] Sharanabasappa S Kulkarni KKA Mallapur CP Gundannavar KP Kambrekar D (2007) Feeding potential of *Ischiodonscutellaris* (Fabricius) (Diptera: Syrphidae) on green peach aphid, *Myzuspersicae* (Sulzer) (Homoptera: Aphididae). Journal of Biological Control 21: 177–178.

[B96] Shukla US Sinha OK (2009) Sugarcane disease: Host-parasite relationship and their integrated management. In: Upadhyay RK Mukerji KG Chamola BP Dubey OP (Eds) Integrated Pest and Disease Management.APH Publishing Corporation, New Delhi, 381–410.

[B97] Singh CAK (1977) *Rhopalosiphumrufiabdominalis* Sasaki, an additional vector of Maize mosaic virus in India. Science and Culture 43(1): 1–37.

[B98] Skvarla MJ Halbert SE Foottit RG Jensen AS Maw E Miller GL (2017) An update to the adventive aphids (Hemiptera: Aphidoidea) of America North of Mexico, with notes on intercepted species. Proceedings of the Entomological Society of Washington 119(1): 90–111. https://doi.org/10.4289/0013-8797.119.1.90

[B99] Skvarla MJ Miller GL Bauchan G Lewis M Foottit R Maw E (2018) Taxonomy and Natural History of Cattail Aphids, *Rhopalosiphumenigmae* (Hemiptera: Aphidomorpha: Aphididae), Including a New Synonymy and Notes on Ant and Parasitoid Associates of *Rhopalosiphum*. Insect Systematics and Diversity 2(2): 1–14. https://doi.org/10.1093/isd/ixy001

[B100] Smith HC (1964) A Survey of Barley Yellow Dwarf Virus in Australia. New Zealand Journal of Agricultural Research 7(3): 239–247. https://doi.org/10.1080/00288233.1964.10416408

[B101] Starý P (1975) *Aphidiuscolemani* Viereck: Its taxonomy, distribution and host range (Hymenoptera, Aphidiidae). Acta Entomologica Bohemoslovaca 72: 156–163.

[B102] Starý P Ghosh AK (1983) Aphid parasitoids of India and adjacent countries (Hymenoptera: Aphidiidae). Zoological Survey of India. Technical Monograph 7: 1–96.

[B103] Sunde RG Maddison PA Tocker PF (1987) Notes on some aphids (Homoptera) from Vanuatu. New Zealand Entomologist 9: 86–88. https://doi.org/10.1080/00779962.1987.9722498

[B104] Tao CC (1963) Revision of Chinese Macrosiphinae. Plant Protection Bulletin of Taiwan 5(3): 162–205.

[B105] Teulon DAJ Stufkens MAW (1998) Current Status of New Zealand Indigenous Aphids. Conservation Advisory Science Notes 216, Department of Conservation, Wellington, 23 pp.

[B106] Teulon DAJ Stufkens MAW (2002) Biosecurity and Aphids in New Zealand. New Zealand Plant Protection 55: 12–17. https://doi.org/10.30843/nzpp.2002.55.3906

[B107] Teulon DAJ Till CM Bennett SJ Green OR Flynn AR Henderson RC Larivière M-C Maw HEL Foottit RG (2010) TFBIS funded specimen information – Aphids. http://www.landcareresearch.co.nz/resources/collections/nzac/tfbis/aphids [accessed on 9 February 2018]

[B108] Teulon DAJ Stufkens MAW Drayton GM Maw HEL Scott IAW Bulman SR Carver M Von Dohlen CD Eastop VF Foottit RG (2013) Native aphids of New Zealand—diversity and host associations. Zootaxa 3647(4): 501–517. https://doi.org/10.11646/zootaxa.3647.4.1 26295124 10.11646/zootaxa.3647.4.1

[B109] Tomanović Z Kavallieratos NG Starý P Stanisavljević LAZ Ćetković A Stamenković S Jovanović S Athanassiou CG (2009) Regional tritrophic relationship patterns of five aphid parasitoid species (Hymenoptera: Braconidae: Aphidiinae) in agroecosystem dominated landscapes of Southeastern Europe. Journal of Economic Entomology 102(3): 836–854. https://doi.org/10.1603/029.102.0302 19610396 10.1603/029.102.0302

[B110] Tomanović Z Starý P Kavallieratos NG Gagić V Plećaš M Janković M Rakhshani E Ćetković A Petrović A (2012) Aphid parasitoids (Hymenoptera: Braconidae: Aphidiinae) in wetland habitats in western Palaearctic: key and associated aphid parasitoid guilds. Annales de la société entomologique de France (N.S.) 48(1–2): 189–198. https://doi.org/10.1080/00379271.2012.10697763

[B111] Turbelin AJ Malamud BD Francis RA (2017) Mapping the global state of invasive alien species: Patterns of invasion and policy responses. Global Ecology and Biogeography 26(1): 78–92. https://doi.org/10.1111/geb.12517

[B112] Van Emden HF Harrington R (2007) Aphids as Crop Pests. CABI Publishing, London, 717 pp. https://doi.org/10.1079/9780851998190.0000

[B113] Vogel R (1978) Compte rendu de mission en Nouvelle-Calédonie du 17 au 22 novembre 1978. IRFA – SRA de San Giuliano, 6 pp.

[B114] Von Dohlen CD Teulon DAJ (2003) Phylogeny and historical biogeography of New Zealand indigenous Aphidini Aphids (Hemiptera, Aphididae): An hypothesis. Annals of the Entomological Society of America 96(2): 107–116. https://doi.org/10.1603/0013-8746(2003)096[0107:PAHBON]2.0.CO;2

[B115] Watanabe S Greenwell AM Bressan A (2013) Localization, concentration, and transmission efficiency of *Banana bunchy top virus* in four asexual lineages of *Pentalonia* aphids. Viruses 5: 758–775. https://doi.org/10.3390/v5020758 23435241 10.3390/v5020758 PMC3640525

[B116] Wegierek P Żyła D Homan A Cai C Huang D (2017) New genus and species of the extinct aphid family Szelegiewicziidae and their implications for aphid evolution. The Science of Nature 104: 1–95. https://doi.org/10.1007/s00114-017-1517-x 10.1007/s00114-017-1517-x 29064069

[B117] Wilson M Evenhuis NL (2007) Checklist of Fiji Auchenorrhyncha and Sternorrhyncha. Fiji Homopteran Checklist, Bishop Museum Technical Report 38(10): 1–24. http://hbs.bishopmuseum.org/fiji/checklists/homoptera.html [accessed on 11 June 2014]

[B118] Work TT Mccullough DG Cavey JF Komsa R (2005) Arrival rate of non indigenous insect species into the United States through foreign trade. Biological Invasions 7: 323–332. https://doi.org/10.1007/s10530-004-1663-x

[B119] Wu GA Terol J Ibanez V López-García A Pérez-Román E Borredá C Domingo C Tadeo FR Carbonell-Caballero J Alonso R Curk F Du D Ollitrault P Roose ML Dopazo J Gmitter FG Rokhsar DS Talon M (2018) Genomics of the origin and evolution of *Citrus*. Nature 554: 311–316. https://doi.org/10.1038/nature25447 29414943 10.1038/nature25447

[B120] Yano K Miyake T Eastop VF (1983) The biology and economic importance of rice aphids (Hemiptera: Aphididae): a review. Bulletin of Entomological Research 73: 539–566. https://doi.org/10.1017/S0007485300009160

[B121] Zimmerman EC (1948) Insects of Hawaii (Vol. I): Introduction. University of Hawaii Press, Honolulu, 206 pp.

